# Unlocking Energy Potential: Exploiting Anionic Redox Activity in Na‐Based Layered Oxides

**DOI:** 10.1002/smll.202512974

**Published:** 2026-03-27

**Authors:** Neeraja Nair, Greeshma Caroline, Rishikesh Vengarathody, Shantikumar V. Nair, Maximilian Fichtner, Prabeer Barpanda, Senthilkumar Baskar

**Affiliations:** ^1^ Amrita School of Nanosciences and Molecular Medicine Amrita Vishwa Vidyapeetham Kochi Kerala India; ^2^ Helmholtz Institute Ulm (HIU) Electrochemical Energy Storage Ulm Germany; ^3^ Institute of Nanotechnology (INT) Karlsruhe Institute of Technology (KIT) Karlsruhe Germany; ^4^ Faraday Materials Laboratory (FaMaL) Materials Research Center Indian Institute of Science Bangalore India

**Keywords:** anionic redox, capacity, energy density, layered oxides, sodium‐ion batteries

## Abstract

Sodium‐ion batteries (SIBs) are progressively recognized as a viable alternative to lithium‐ion batteries due to their operational and economic viability. However, their practical application is limited by lower energy density and intermediate cycling performance, predominantly limited by the cathodes. The exploration of anionic redox in layered oxides has introduced a new approach to enhance the energy density of rechargeable Na‐ion batteries. By exploiting anionic redox activity, various positive electrodes are capable of providing additional capacity beyond their theoretical capacity. This additional capacity emerges from the combined contributions of anionic and cationic redox processes. However, the anionic capacity achieved during charge is often only partially reversible upon discharge, posing a significant challenge for practical use. This review offers a comprehensive analysis of anionic redox phenomena in sodium‐based layered oxides, summarizing recent research strategies aimed at improving the anionic and cationic redox performance of these cathode insertion materials. It also elucidates the relationship between structure, function, and electrochemical performance. Eventually, it provides insights into the future research directions for Na‐based layered oxide cathodes for energy storage applications.

## Graphical abstract

Anionic redox in Na‐based layered oxide cathodes unlocks additional capacity beyond conventional transition‐metal redox limits, enabling high‐energy sodium‐ion batteries. This review elucidates coupled cationicanionic redox mechanisms across P_2_‐, O_3_‐, and related structural phases, while critically addressing key challenges such as voltage hysteresis, oxygen loss, and structural degradation. It further highlights material design strategies and advanced characterization techniques (mRIXS, XAS, EPR, and EELS) to establish structurepropertyperformance relationships, guiding the development of stable, high‐performance Na‐ion battery cathodes

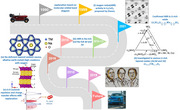

## 1 Introduction

Developing economic and efficient energy storage systems is crucial for enhancing the stability and feasibility of utilizing renewable energy resources, rivalling traditional fossil fuel‐based power generation [1, 2, 3]. As technology continues to progress, renewable energy conversion and storage hold a potential solution to cater to micro‐to‐mega scale applications [4, 5, 6]. Among the commercially available energy storage systems, lithium‐ion batteries (LIBs) are indeed a mature and extensively employed technology in a plethora of applications, including vehicle propulsion, stationary energy storage, and portable electronic devices [7, 8, 9, 10, 11, 12]. However, the scarcity of lithium resources, uneven distribution, and potential interruptions of the supply chain have led to the exploration of alternative battery technologies. At this juncture, sodium‐ion batteries (SIBs) have emerged as sustainable alternate to LIBs owing to their following advantages: (i) ample Na resources (23000 ppm in Earth's crust), (ii) feasible extraction and distribution of Na from seawater, (iii) usage of cobalt‐free layered oxide cathodes, (iv) operational safety allowing transportation of SIBs in zero‐volt discharged state, (v) similar (*rocking chair*) working mechanism, (vi) use of low‐cost Al‐foil as both anode and cathode current collector and (vii) drop in technology—low CapEx [13, 14, 15, 16, 17, 18, 19, 20, 21]. These characteristics make them well‐suited for large‐scale electrical energy storage applications, e.g., in electrical grids. Each component of sodium‐ion batteries has its own advantages and challenges, which provide impetus for steady exploration of new materials for improved battery performance. In this quest, the cathode materials can play a pivotal role in making high‐energy‐density SIBs more competitive with other energy storage technologies [22, 23, 24, 25, 26]. Suites of layered oxides, polyanionic compounds, Prussian blue analogues, and organic materials have been explored as cathode active materials [27, 28, 29, 30, 31, 32]. Among them, layered transition metal (TM) oxides have been widely employed in commercial LIB technology, and this trend holds true for sodium‐ion batteries as the layered oxides offer high capacity, scalable synthesis, and high gravimetric and volumetric energy density [33, 34, 35, 36, 37, 38].

**FIGURE 1:**
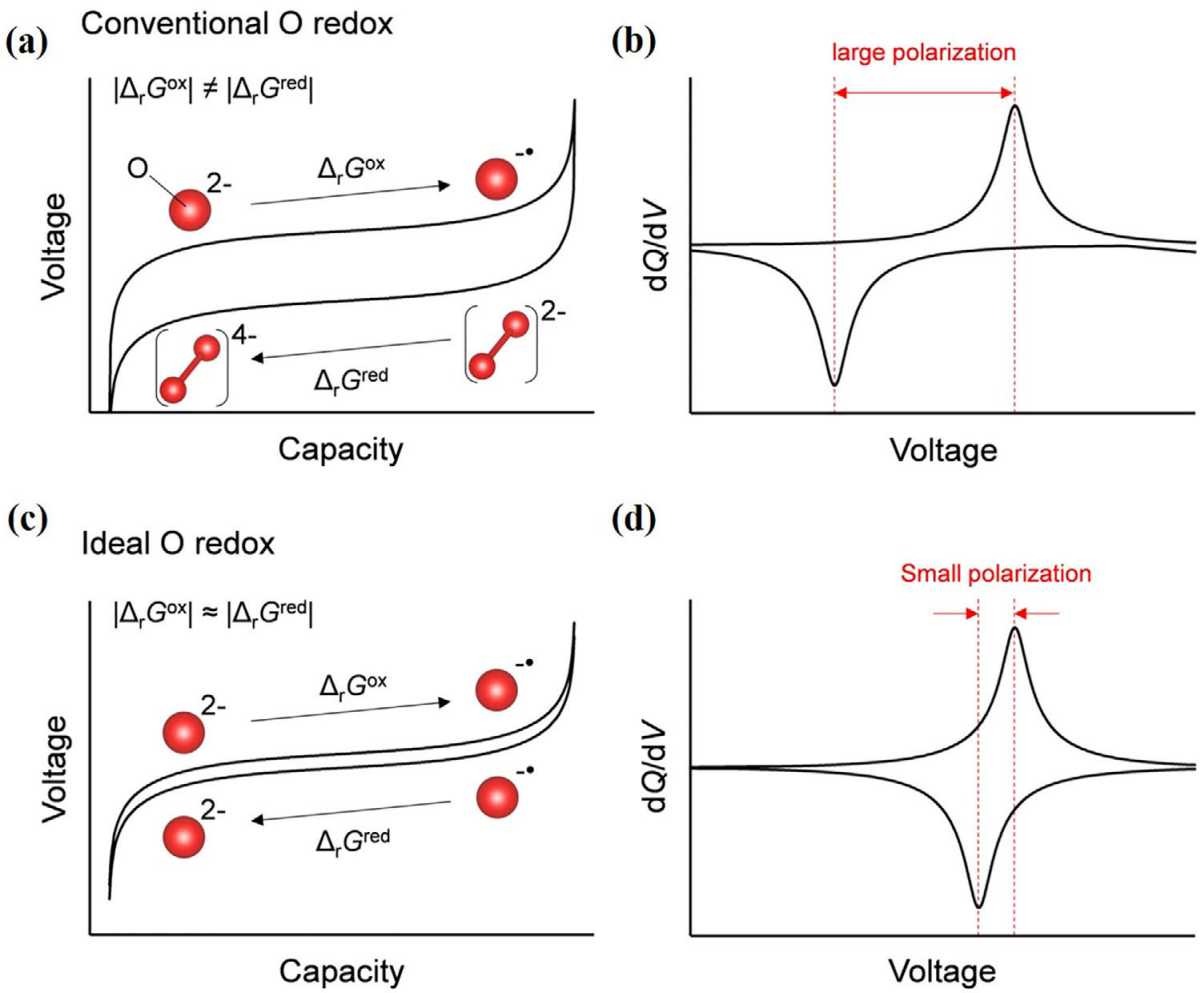
Schematic illustration of charge/discharge curves and d*Q*/d*V* plots (*Q*: specific capacity, *V*: reaction voltage) for (a and b) conventional oxygen redox with large polarization (O^2−^/O_2_^2−^), and (c and d) ideal oxygen redox with small polarization (O^2−^/O^−•^). The red sphere denotes oxygen atoms [56]. Reproduced from reference [56] 2021 Nature Communications CC‐BY‐4.0.

Contrary to Li_x_TMO_2_ (O3‐type Li‐based layered oxides), sodium‐based layered transition oxide (Na_x_TMO_2_) cathodes can indeed be classified into three main categories based on the Na environment and oxygen stacking sequences: Na‐rich, Na‐full (O3), and Na‐deficient oxides [39, 40, 41, 42]. The Na‐deficient layered oxides are divided into P2, P3, and O2‐type as per Delmas notation, where P and O denote prismatic and octahedral sites, respectively [43]. For instance, P2 and O2 represent two layers of stacking, while O3 and P3 represent three layers of distinct oxygen stacking within a unit cell. Sodium ions in P2 and P3 demonstrate a direct prismatic diffusion pathway facilitating high‐rate capability. However, the initial lower sodium content limits their storage capacity. In contrast, O3 configurations offer higher storage capacity due to full sodium content, albeit with lower rate capability owing to off‐pathway Na^+^ migration mechanisms, wherein Na^+^ transport deviates from the conventional octahedral‐to‐octahedral hopping route [33]. These materials exhibit low capacity. Thus, extending the voltage ranges is crucial to trigger oxygen ion participation in the redox process to increase capacity. However, the oxygen redox involvement may or may not be reversible [44, 45, 46]. Oxygen redox often have effects such as structural instability, oxygen loss, and voltage decay [46]. Nevertheless, the interactions between Na and O (Na‐O‐Na) in Na‐rich, Na‐full (O3), and Na‐deficient oxides stemming from the formation of nearly non‐bonding oxygen states stimulate oxygen redox behavior, providing extra capacity [47, 48, 49, 50, 51]. Oxygen redox activity is a key area of current battery research. Elucidating the mechanisms of oxygen‐based redox processes holds significant promise for next‐generation batteries. During Na^+^ deintercalation from the cathode structure, the Fermi level shifts to a lower energy due to the oxidation of TM. As a result, it approaches the energy level of the O2*p* orbitals, creating a high‐energy non‐bonding state of oxygen. This state facilitates the anionic redox reaction, leading to the oxidation of *O* ^2 −^ to *O* ^−^ ions, often referred to as superoxide [52, 53, 54, 55]. When the Gibbs energies for oxidation and reduction are comparable, the charge and discharge processes (*O* ^2 −^ ↔ *O* ^−^) are reversible that would proceed with minimal energy loss (Δ_*r*_ *G^ox^* ≈ Δ_*r*_ *G^re^*)  [56] Attaining this reversible intercalation of sodium involves oxygen anions that remain lattice‐bound and undergo redox without detachment is pivotal for the whole battery system as it enhances both efficiency and performance. Consequently, small polarization signifies that the voltage drops across the battery during charging and discharging is negligible, promoting sustained high performance across multiple cycles (Figure 1 a and c). Unlike earlier processes, where the oxidation and reduction free energies $\left(\Delta_{r}G^{ox}\neq \Delta_{r}G^{re}\right)$) are distinct, anionic redox reactions do not terminate at the *O* ^2 −^ /*O* ^−^ couple. Instead, they proceed further to form *O* ^2 −^ /$O_{2}^{n-}$ n‐(peroxo‐like dimers) along the c‐axis. This leads to the formation of unstable O–O dimers, which eventually evolve as molecular O_2_, contributing to structural degradation.

As a consequence, oxygen loss from the lattice leads to oxygen vacancies. The large voltage hysteresis observed in the dQ/dV peaks during charge/discharge processes (Figure 1b,d) further supports the occurrence of these irreversible reactions, especially those involving oxygen [56, 57, 58, 59, 60]. Moreover, irreversible release of oxygen leads to a densification of the material and accelerates the formation of cracks on the surface of the cathode, triggering TM dissolution, Na^+^ dissolution, and side reactions resulting in unstable cycling stability. As a consequence, this hampers efficient Na^+^ ion transfer kinetics.

Tarascon et al. demonstrated that by harnessing both anion and cation redox reactions, it is possible to design materials that could potentially increase the overall capacity of batteries [61]. Ren et al. explored the relative anionic redox for Na‐based layered oxides across 3*d*, 4*d*, and 5*d* transition metal series [62]. This has led to research focused on increasing the energy density of these materials. However, these efforts often concentrate on optimizing the structures, tackling challenges related to cycling stability, capacity retention, and structural alterations. Chen et al. proposed a comprehensive and systematic methodology for tailoring electrodes in LIBs and SIBs. This approach particularly emphasizes on the strategic incorporation of nitrogen doping or substitution into electrode materials [63]. Such strategic integration of anionic redox activity could significantly enhance the overall performance and efficiency of batteries. These perspectives collectively enrich the ongoing efforts aimed at enhancing the efficacy and viability of energy storage systems. Several reviews delve into the fundamental aspects, future potential, and relevance of anionic redox in batteries [5, 64, 65, 66, 67].

Herein, based on recent literature, this review discusses the fundamental aspects of anionic redox with a primary focus on sodium‐layered oxide cathodes. We first summarize the key mechanistic features governing anionic redox and its coupling with cationic redox during electrochemical cycling. Subsequently, recent material strategies reported for stabilizing anionic redox in Na‐based layered oxides are systematically discussed, with emphasis on compositional tuning, electronic‐structure modulation, and redox coupling concepts. Advances in experimental and computational characterization techniques used to probe anionic redox and associated structural evolution are then reviewed. Finally, the remaining challenges are outlined together with possible strategies, aiming to provide clear guidance for the rational design of sodium‐ion battery cathodes involving reversible anionic redox.

## 2 Unveiling the Journey: Exploring the Evolution of Anionic Redox

**FIGURE 2:**
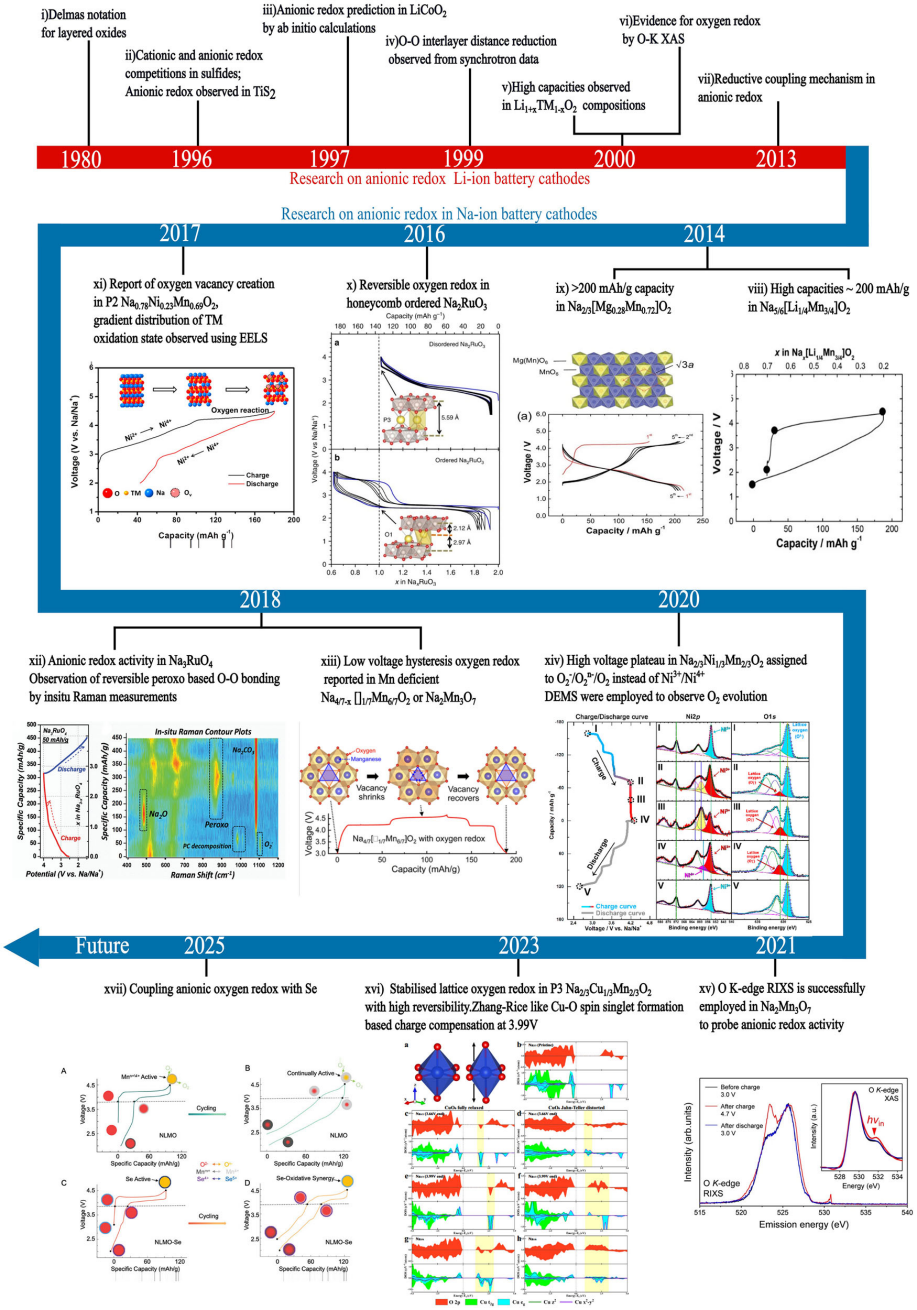
Schematic roadmap illustrating key developments in anionic redox materials and characterization approaches. Images corresponding to different years are reproduced with permission from the respective references: (viii).Estimated Na contents from the oxidation/reduction capacity (Na_x_[Li _1/4_ Mn _3/4_]O_2_) [275] Reprinted with permission [275] 2014 WILEY‐VCH Verlag GmbH Co. KGaA, Weinheim.; (ix). Electrochemical characterization of Na_2/3_[Mg_0.28_Mn_0.72_]O_2_ and structure [276] Reprinted with permission [276] 2014 journal of European chemistry, All rights are reserved; (x). Galvanostatic cycling curves recorded at 30 mA g^−1^ for disordered and ordered Na_2_RuO_3_ [177] Reprinted with permission [177] 2016 CC‐BY‐4.0. (xi).Charge compensation mechanisms in the P2− Na_0.78_Ni_0.23_Mn_0.69_O_2_ cathode [277] Reprinted with permission [277] 2017, American Chemical Society. (xii).voltage profile and capacity dependent in situ Raman spectral contour plots [181] Reprinted with permission [181] 2018, Royal Society of Chemistry. (xiii).Structure and GCD curves of Na_4/7_Mn_6/7_⬜_1/7_O_2_ [247] Reprinted with permission [247] 2019, American Chemical Society. (xiv).GCD curves of Na_2/3_Ni_1/3_Mn_2/3_O_2_ (0.1 C) and Ni 2p/O 1s XPS at selected charge states [278] Reprinted with permission [278] 2020 American Chemical Society. Published under ACS AuthorChoice License.(xv). O K‐edge RIXS [56] Reproduced from reference [56] 2021 CC‐BY‐4.0, Published under Nature AuthorChoice License.(xvi).DFT‐calculated pDOS of P3‐type Na_2/3_Cu_1/3_Mn_2/3_O_2_ at different desodiation state [279] Reprinted with permission [279] 2023 CC‐BY, Published under Nature AuthorChoice License. (xvii).Schematic of reductive couple mechanism [252] Reprinted with permission [252] 2024 WILEY‐VCH Verlag GmbH Co. KGaA, Weinheim.

The revelation of the anionic redox process emerged alongside the development of LIBs, catalyzing research and innovation in energy storage solutions [61, 62]. Despite the exceptional performance of LIBs, researchers continually attempt to improve energy density by expanding capacity through extending the operating voltage window [68, 69, 70]. Extending the voltage window brings the oxygen redox process into the spotlight, which presents both advantages and challenges for practical applications. Hence, a comprehensive study is required to understand the redox mechanisms and to utilize the advantages of oxygen redox processes in battery technologies while mitigating the associated challenges. This phenomenon was first depicted using a band diagram in the early 1990s, illustrating the effects of hybridization and splitting that result from the interactions of various *s*, *p*, and *d* atomic orbitals [71, 72, 73]. It reveals how oxygen atoms participate in bonding [74, 75] as well as how electrons occupy distinct orbitals within the material [76, 77]. To further understand and demonstrate how this phenomenon influences capacity, Li‐rich TM oxide cathode materials such as LiCoO_2_ and Li [Ni_x_Co_y_M_1‐x‐y_]O_2_ have been initially studied. These materials exhibit good electrochemical performance and high energy density, which makes them prominent. Figure 2 illustrates the timeline of the development and exploration of anionic redox in Na‐based layered oxide cathodes.

The inception of oxygen redox activity in Li*_x_* CoO_2_ was rationalized in the 1990s, based on the observation of a slight reduction in O–O distances as determined by synchrotron diffraction, which was further supported by magnetic study [78, 79, 80, 81, 82, 83, 84, 85, 86, 87, 88]. Afterward, it was discovered that adding excess lithium to a layered LiTMO_2_ structure (such as $Li(Ni_{x}Li_{\left(\frac{1}{3}-\frac{2x}{3}\right)}Mn_{\left(\frac{2}{3}-\frac{x}{3}\right)}$)*O*_2_) shows an extra plateau at high voltage which can increase the materials capacity to 220 mAh g^−^^1^. This additional capacity may result not only from oxygen redox, but also from Mn oxidation beyond the 4^+^ state or Li^+^/H^+^ electrolyte decomposition, which compensates for the additional lithium deintercalation [79, 89, 90, 91, 92, 93, 94, 95, 96, 97]. Subsequently, multiple Li‐rich layered phases, like *Li*_1 + *x*_ *TM*_1 − *x*_ *O*_2_, were documented in 2002 to demonstrate notably high capacity along with the redox activity of transition metals and an extended voltage plateau at high voltage. For instance, confederation of Li_2_MnO_3_ and LiMO_2_ end‐members can be used to depict these materials on a ternary phase diagram [98, 99, 100, 101, 102, 103]. Based on the investigations carried out by several research groups regarding early claims of electrochemical activity in Li_2_MnO_3_, high‐capacity lithium‐rich NMC (Nickel Manganese Cobalt) compounds were discovered [104, 105]. Following suites of characterizations on Li‐rich NMC compounds, the reversible redox activity of lattice oxygen was verified [106].

The emergence of higher energy *d* orbitals (4*d* and 5*d*) promotes stronger covalent bonding with oxygen anions due to extensive orbital overlap as observed in the case of Li_2_RuO_3_ [107, 108, 109] and Li_2_IrO_3_ [110, 111, 112, 113]. As new materials emerge, the development of characterization methods is crucial for enhancing and exploiting their properties effectively. Recently, theorists have verified the previously described oxygen activity concurrently using electron density map calculations. To evaluate the oxygen redox activity, various spectroscopic measurements of the electronic state of the ligand oxygen were conducted, including O 1*s* XPS (X‐ray photoelectron spectroscopy) and O K‐edge XAS (X‐ray absorption spectroscopy) [114, 115, 116, 117, 118, 119].

While 4*d* and 5*d* cathode materials have been widely studied for enhancing cationic and anionic redox in Li‐ion batteries, their potential in Na*x*TMO_2_ is being explored to improve performance in sodium‐ion batteries. Currently, 4*d* and 5*d* cathode materials are being investigated in order to promote the cationic and anionic redox reactions in Na*_x_* TMO_2_. However, anionic redox is an area of active research, and its application comes with challenges related to the stability of electrode materials and the electrolytes [120, 121, 122].

The discovery of additional capacity contributed by lattice oxygen ions highlights the complex electrochemical behavior of layered oxide cathode materials [123, 124, 125, 126]. It opens up possibilities for optimizing sodium‐ion battery performance by utilizing the unique characteristics of Na‐based layered oxides and broadening insights into their electrochemical dynamics [127, 128, 129]. Initially, Na‐rich and Na‐full 3*d*(e.g. Mn) metal layered oxides are used due to their high capacity, affordable cost, and elemental abundance with capacities exceeding 250 mAh g^−1^. However, oxygen release during charging and cation movement inside the crystal lattice leads to voltage degradation and irreversible capacity fading during cycling [129, 130, 131, 132].

On the other hand, compounds utilizing 4*d* (e.g., Ru) [123, 131, 133, 134, 135, 136] and 5*d* (e.g. Ir) [137, 138, 139, 140, 141] noble metals have attained prominent interest. Studies have indicated that transitioning from 3*d* (Mn) to 4*d* (Ru)/5*d* (Ir) metals can enhance transition metal‐oxygen (TM‐O) covalency, consequently stabilizing oxygen‐redox reactions. Increasing structural integrity, which improves the covalency, reduces stress from the structure related to the extraction of Na‐ions. Indeed, the preceding observations pave the way for the application of anionic redox chemistry in Na‐deficient layered oxides [123, 133, 134, 135, 136, 137, 138, 139, 140, 141]. Anionic oxygen redox reaction offers options to investigate high electrochemical performance cathodes by enhancing energy density and delivering higher capacity. Investigating the basic mechanism of anionic redox is necessary to advance its potential commercial applications. Until now, various groups have used theoretical and computational analysis to uncover the anionic redox process. The next section presents the fundamental mechanistic insights pertaining to anionic redox activity [142].

## 3 Mechanistic Insights Into the Anionic Redox Reaction

A detailed understanding of the anionic redox process is vital for advancing its commercial viability. Despite extensive theoretical studies, its complete mechanistic basis remains unresolved due to electronic–structural coupling. This section presents a unified discussion of the electronic factors governing anionic redox, emphasizing the role of oxygen *sp* and transition‐metal *d* orbitals in charge compensation.

Anionic redox in layered oxides arises from partial overlap of transition‐metal *d* and oxygen *sp* orbitals, enabling charge compensation [25, 32, 142, 143]. In cathodes, during desodiation, electrons are extracted from orbitals situated below the Fermi level; partial TM 3*d–*O 2*p* overlap facilitates the participation of oxygen 2*p* bands during this process [145, 146, 147, 148, 149]. Hybridization of TM 3*d* and O 2*p* orbitals forms bonding, antibonding, and nonbonding states that govern redox reversibility [125, 150, 151, 152, 153]. TM 4*s* and 4*p* orbitals further participate in hybridization, modulating the electronic pathways for oxygen redox. These electronic interactions were systematically rationalized by Rouxel and co‐workers using a schematic band‐structure framework. This framework correlates the Fermi level (E_F_) with electrochemical redox potential, governing anionic redox behavior [71, 73].

**FIGURE 3:**
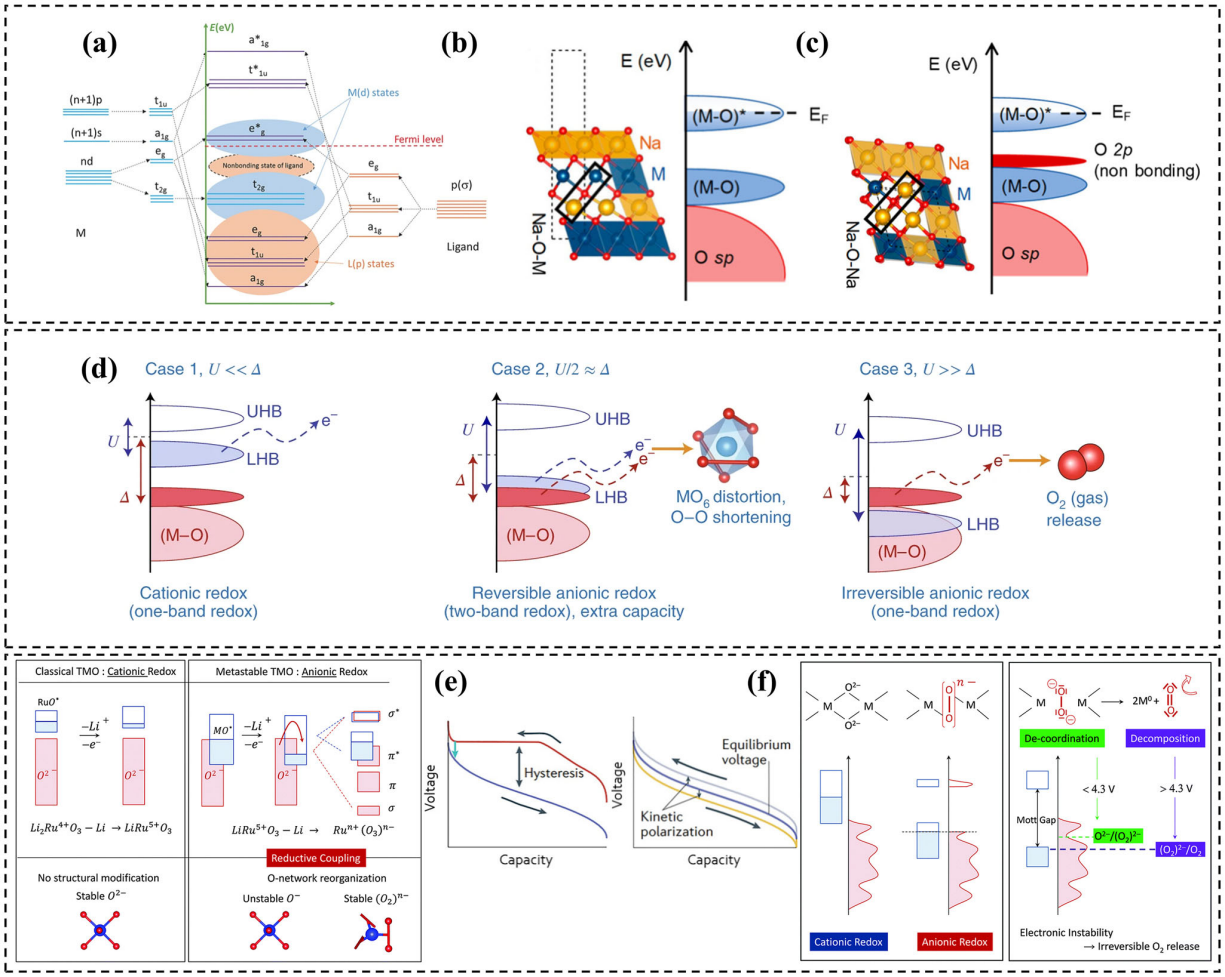
(a) Molecular orbital energy level diagram between transition metal and oxygen 2*p* [125]. Reprinted with permission [125] 2017 WILEY‐VCH Verlag GmbH & Co. KGaA, Weinheim (b) and (c) Schematic structure and electronic band diagram of Na‐based layered oxide [59]. Reprinted with permission [59] CC‐BY‐4.0. (d) The upper and lower Hubbard bands are designated as UHB and LHB. The overlap of LHB and non‐bonding O 2*p* states depending on how the d‐d Coulomb repulsion term U and the charge transfer term Δ interact [61]. Reprinted with permission [61] CC‐BY‐4.0. (e) Schematic band structure illustrating cationic versus anionic redox processes in transition‐metal oxides with strong M–O covalency. (f) When O_2_ gas release occurs in the first charge due to cationic migration/disorder, the process is not reversible, leading to a persistent hysteresis in further cycles and an S‐shape curve due to the multiplication of transition metal and (O–O) redox centres [143, 144]. Reprinted with permission [143, 144] 2022, Springer Nature Limited, All rights reserved.

Figure 3a presents a TM–O molecular‐orbital diagram, showing σ‐overlap between transition‐metal *d* and oxygen *sp* orbitals, illustrating energy levels [154]. The diagram indicates the relative positions of occupied and unoccupied states that collectively describe anionic redox behavior. Partial overlap of TM 3*d* and O 2*p* orbitals forms bonding (M− O), antibonding (M− O)*, and nonbonding states in the TMO_6_ octahedron. The five 3*d* orbitals (d_x_^2^_−y_^2^, d_z_^2^, d_xy_, d_xz_, d_yz_) are split into *e_g_* (d_x_^2^_‐y_^2^, d_z_^2^) and *t_2g_* (d_xy_, d_xz_, d_yz_) levels [132, 152, 155, 156, 157]. The linear combination of atomic orbitals generates bonding and antibonding molecular orbitals, where the occupancy of antibonding orbitals weakens bonds. This modulation of orbital occupancy affects molecular reactivity and determines the reversibility of anionic redox processes [61, 62, 143, 158, 159].

Figure 3b,c extends the molecular‐orbital concept to real Na‐layered oxide systems, illustrating how weak Na–O interactions modulate oxygen participation in redox. Weak interactions (Na–O–Na and Na–O–TM) reduce the covalent character of certain O 2*p* orbitals, resulting in near nonbonding oxygen states. These weakly bound oxygen orbitals lie adjacent to the Fermi level, making them energetically favourable sites for electron removal during charge—discharge cycles. Consequently, electrons can be reversibly extracted from oxygen without destabilizing the TM–O framework, providing the mechanistic basis for reversible anionic redox [59, 62]. This phenomenon is ascribed to the oxygen evolution in a state that is nearly non‐bonding, thereby facilitating its involvement in the redox processes.

Building on the Zaanen–Sawatzky–Allen classification/Mott–Hubbard and the unified framework proposed by Rouxel and co‐workers, the following discussion places these orbital‐level descriptions into a broader electronic‐structure context, explicitly accounting for electron correlation effects and band‐energy alignment during alkali‐ion extraction [160, 161, 162]. The electrochemical activity of alkali‐rich transition metal oxides is fundamentally governed by their electronic ground state, which is defined by the relative energies of transition metal *d* states and oxygen 2*p* states. In layered TM oxides, strong hybridization between metal *d* orbitals and oxygen *p* orbitals leads to the formation of bonding (M− O) and antibonding (M− O)* states. The antibonding (M–O)* band is typically partially filled and lies close to the Fermi level, while the oxygen 2*p* band remains fully occupied at lower energy. The energy separation between these states is described by the charge‐transfer energy (Δ), whereas the on‐site Coulomb repulsion between *d* electrons is represented by the Hubbard parameter (U) [163].

In systems where electron–electron interactions are significant, the partially filled (M− O)* band does not remain continuous. Instead, localized Coulomb repulsion splits this band into two distinct components: the lower Hubbard band (LHB), which is occupied, and the upper Hubbard band (UHB), which is unoccupied. This splitting reflects the energetic penalty associated with placing two electrons on the same transition metal site. The magnitude of U depends strongly on the spatial extent of the *d* orbitals and therefore decreases from 3*d* to 4*d* and 5*d* transition metals due to increased orbital delocalization.

During alkali‐ion extraction, such as Na^+^ or Li^+^ deintercalation, the local electrostatic environment around oxygen is significantly altered. The removal of positively charged alkali ions destabilizes nearby oxygen 2*p* states, shifting them upward in energy, while the transition metal–derived states experience electrostatic stabilization. As a result, the relative positions of the LHB and oxygen 2*p* bands evolve dynamically during charging. In classical Mott–Hubbard systems (*U* ≪ Δ), the LHB remains energetically closer to the Fermi level than the oxygen 2*p* band, and electron removal proceeds predominantly from transition metal states. This corresponds to conventional cationic redox and is generally reversible.

However, when U and Δ become comparable, the energetic separation between the LHB and the oxygen 2*p* band is significantly reduced. In this regime, small perturbations induced by alkali‐ion removal can bring these states into near degeneracy. Such an electronic configuration is inherently unstable and cannot be sustained without structural relaxation. To lift this degeneracy, the lattice undergoes local symmetry breaking, typically through distortions of the MO_6_ octahedra and reorganization of the oxygen sublattice. This structural response enables effective interaction between transition metal and oxygen states that were previously symmetry‐forbidden. As a consequence, electron density is partially transferred from oxygen 2*p* orbitals into newly formed bonding configurations involving neighbouring oxygen atoms, leading to the formation of O–O dimers.

This process, often described as reductive coupling, results in the emergence of (*O* − *O*)^*n* −^ species and marks the activation of anionic redox. Importantly, in this scenario, oxygen redox is not an independent process but is dynamically triggered by the instability of the cationic electronic structure. The transition metal initially oxidized can even undergo partial reduction during lattice relaxation, highlighting the coupled nature of cationic and anionic redox.

In charge‐transfer–dominated systems (*U* ≫ Δ), the oxygen 2*p* band lies closer to the Fermi level than the LHB even before extensive alkali extraction. Upon charging, electrons are removed directly from oxygen states, generating highly oxidized and unstable oxygen species. To satisfy electronic stability, these oxygen holes localize through O− O pairing, forming peroxide or superoxide species depending on the degree of oxidation. The stability of these species is determined by the position of their antibonding σ* and π* states relative to the transition metal *d* band. If these oxygen‐derived states lie below the metallic band, the anionic redox process can be reversible. Conversely, if they cross above the metallic band, reductive elimination occurs, leading to molecular O_2_ release, voltage hysteresis, and irreversible capacity loss [61, 62, 160, 161, 162, 163, 164].

Thus, anionic redox is directly related to the evolution of the electronic band structure upon alkali‐ion extraction. The splitting of the (M–O)* band into lower and upper Hubbard bands and the upward shift of oxygen 2*p* states together govern whether oxygen contributes reversibly to charge compensation or leads to irreversible oxidation. This understanding helps explain why materials with moderate TM–O covalency, lower effective U values, and controlled local coordination environments more readily stabilize anionic redox without significant oxygen loss [76].

The effectiveness of alkali–oxygen configurations in triggering anionic redox is strongly dependent on the local chemical environment around oxygen. Among these, Na–O–Na configurations are the most effective in elevating oxygen 2*p* states toward the Fermi level, as the absence of transition‐metal coordination strongly suppresses TM–O hybridization and renders oxygen nearly nonbonding. In contrast, Na–O–TM environments retain partial covalency, resulting in a more moderate activation of oxygen redox and improved reversibility. Importantly, Na–O–X configurations (X = Li, Mg, Zn, Cu, or vacancy) provide an intermediate regime, where ionic X–O interactions selectively weaken TM–O covalency without fully isolating oxygen states. Such configurations enable controlled activation of anionic redox while mitigating excessive oxygen oxidation and structural degradation. These distinctions highlight how targeted cation substitution and vacancy engineering can be used to tune the balance between reversible oxygen redox and lattice stability [76, 161].

Figure 3e,f illustrates how local coordination environments influence the activation of anionic redox, using Ru‐based systems as a representative example. Oxygen redox is initiated by strong overlap between Ru *d* orbitals and O 2*p* states, which destabilizes the oxygen electronic structure during Na extraction. This destabilization induces a reorganization of the oxygen network, leading to the formation of either unstable O^−^ species or more stable (*O*_2_)^*n* −^ units. Oxygen ions in vacancy‐rich environments, such as Na–O–Na or Na–O–X configurations, are particularly susceptible to oxidation. Excessive oxidation can trigger partial reduction of neighbouring Ru ions and O_2_ release, resulting in voltage hysteresis and incomplete capacity recovery during discharge [143, 144].

## 4 Expanding Horizons: Employing Anionic Redox in Layered Oxides

### 4.1 Na‐Rich Layered Oxides for Sodium‐Ion Batteries

Lithium‐rich layered oxide cathodes such as Li_2_MO_3_ (M = Mn, Ru, etc.) can provide capacity over 280 mAh g^−1^ that is higher than the capacity stemming from purely cationic redox (transition‐metal content alone). This extra capacity arises from the oxygen redox process (O^2−^/O^n^), which was first reported in Li‐rich layered oxides, e.g., Li_2_RuO_3_, Li_1.2_Ni_0.2_Mn_0.6_O_2_, Li_2_Ru_0.5_Sn_0.5_O_2,_ and Li_1.3_Mn_0.4_Nb_0.3_O_2_ [50, 165, 166, 167]. The successful investigation of anionic redox performance in Li‐rich manganese‐based layered oxide cathodes (Li_2_MnO_3_) has drawn a lot of interest in the Na analogue Na‐rich Na_2_MnO_3_.

The Na‐rich layered oxides (Na_2_MO_3_) can crystallize into three different crystal structures: O3, O1, and O1′ with the space group of *C*2/c or *C*2/*m*. The O3 type Na‐rich Na_2_MnO_3_ has an octahedrally coordinated oxygen sublattice with ABCABC stacking sequence. On the other hand, O1 has a hexagonal stacking sequence of ABAB. O1′ is another type of structure that forms during the charging process due to the sliding of TM layers [80]. Among these three structures, it is anticipated that the O1′ phase triggers the anionic redox reactions in Na‐rich layered oxides.

The anionic redox properties, transition metal migration, and electrochemical phase stability of 38 Na_2_MO_3_ layered oxides (M = 3*d*, 4*d*, and 5*d* transition metals, TMs and post‐TMs) have been studied by first‐principles calculations [80]. The work predicted that the Na_2_MO_3_ with M = Tc, Ru, Rh, W, Ir, Pt, Mo, and Pd can exhibit high energy density (up to 747 Wh kg^−1^) and stable phase transitions (O3↔O1′↔O1) during (de)intercalation of Na^+^. The Mn‐based sodium‐rich cathodes are promising in terms of high theoretical capacity and materials economy.

Na‐rich cathodes with chemical formula Na_2_MO_3_ and Na_2_RuO_4_ (3*d*, M = Mn, Ti, V; 4*d*, M = Ru, and 5*d*, M = Ir) are explored for their anionic redox properties. Na_2_MO_3_ with 4*d* and 5*d* transition metals, such as Na_2_RuO_3_ and Na_2_IrO_3,_ showed excellent reversible anionic and cationic redox properties. However, the research progress on Na‐rich layered oxide cathodes with 3*d* transition metals are timely important due to the low cost and practical viability. Here, the recent advancements in anionic redox activities of the 3*d*, 4*d*, and 5*d* transition metal‐based Na‐rich cathodes are discussed.

#### 4.1.1 3*d* Transition Metal‐Based Layered Oxides

The Li_2_MnO_3_ showed anionic redox activity at higher voltage due to the presence of Mn^4+^, which could not be oxidized further. The Li_2_MnO_3_‐based Li‐rich oxide cathodes demonstrated excellent anionic redox activity [168]. Following the Li‐rich composition, Na‐analogue Na_2_MnO_3_ was attempted as 3*d* Na‐rich cathode. Theoretical works predicted that the Mn‐based cathode can be a promising anionic redox active cathode that can provide a high capacity of ∼270 mAh g^−1^ [169, 170]. However, the experimental results showed that the anionic redox in the structure is not active due to the ionic radii mismatch of Na^+^(1.02 Å) and Mn^4+^ (0.53 Å) [171]. However, Na‐rich layered oxide derived from 0.5Li_2_MnO_3_•0.5LiMn_0.42_Ni_0.42_Co_0.1_O_2_ by electrochemical method has been demonstrated as high‐capacity cathode (234 mAhg^−1^) with an energy density of 644 Wh kg^−1^ [172].

**FIGURE 4:**
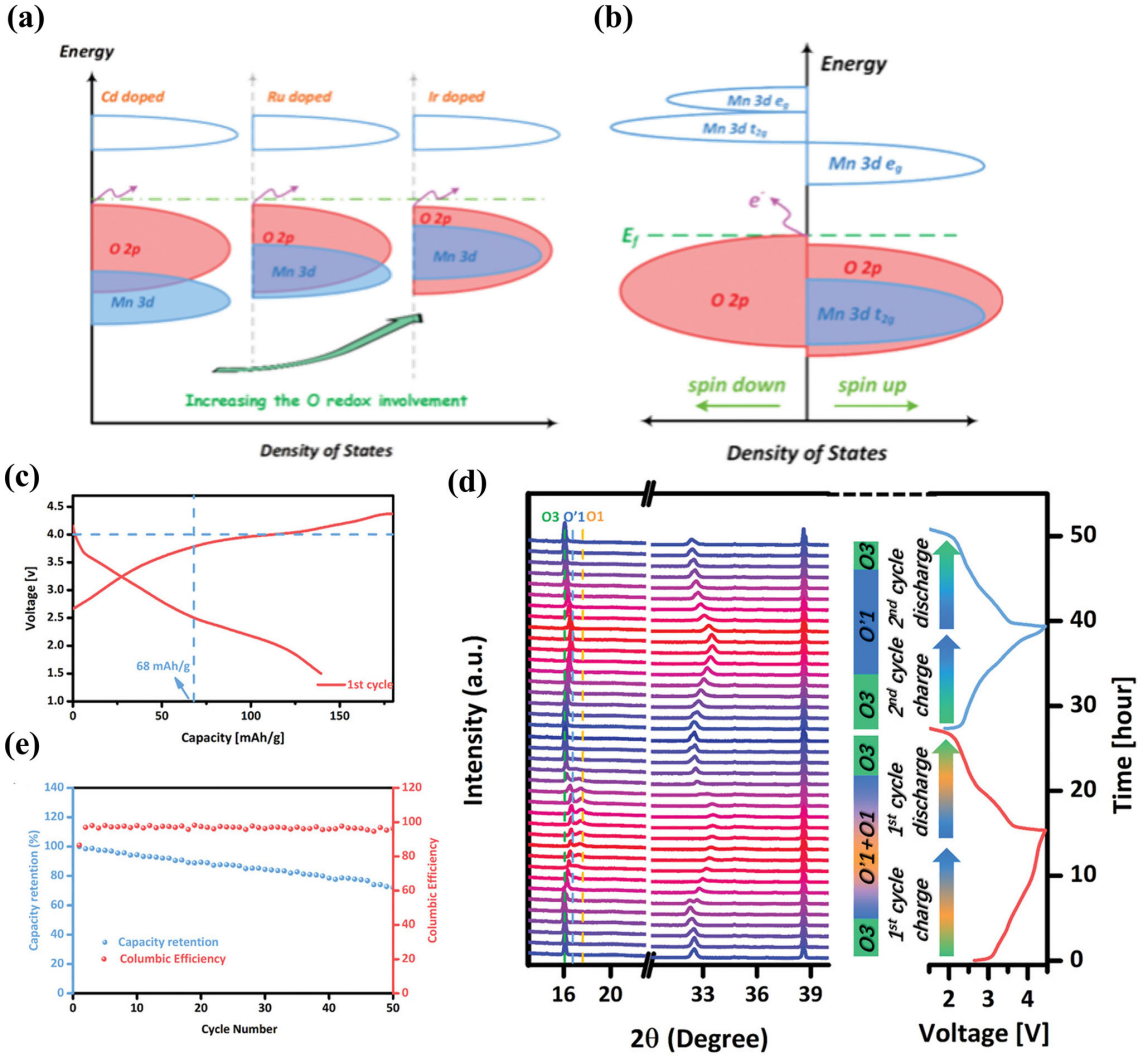
Schematic illustration of the DOS for (a) dopants (Cd, Ru, and Ir) and (b) Ir‐doped Na_1.2_Mn_0.4_Ir_0.4_O_2_ (NMI). The O2*p* and Mn 3*d* bands are depicted in red and blue, respectively. The Cd, Ru and Ir doped Mn‐based Na‐rich systems have a tendency for increasing O redox activity as the Mn 3*d* band increases, which refers to a suppression of O_2_ release. (c) Electrochemical performance of NMI in the first cycle. (d) Cycling performance of NMI. (e) Left: *Operando* XRD patterns collected at a constant current rate of 20 mA g^−1^. Middle: Schematic of phase evolution upon cycling, where the pristine O3 phase, O1′ and O1 coexisting biphase and O1′ phase areas are depicted in green, orange with blue and blue color bars, respectively. Right: the corresponding electrochemical performance during XRD measurement [173]. Reprinted with permission [173] 2019 WILEY‐VCH Verlag GmbH & Co. KGaA, Weinheim.

While limited anionic redox activity is observed for Na_2_MnO_3_, TM‐substituted Na‐rich cathodes exhibits oxygen redox activity. For instance, Na‐rich Na_1.2_Mn_0.4_Ir_0.4_O_2_ (NMI) cathode has been reported as cationic and anionic redox active cathode [173]. The density of states (DOS) obtained from DFT calculations indicate that Ir substitution enhances anionic redox activity compared with Cd‐ and Ru‐substituted systems (Figure 4a). As shown in Figure 4b, the Mn 3*d* states are embedded within the O‐2*p* band with an upward shift in energy, reflecting enhanced Mn–O covalency. This electronic structure stabilizes high‐energy oxygen states and suppresses irreversible O_2_ evolution, enabling reversible anionic redox. The voltage profiles and cyclic performance of NMI (Figure 4c,d) exhibit combined Mn^4+^/Mn^3+^ cationic redox (∼68 mAh g^−1^) and oxygen redox corresponding to ∼0.6 mol Na extraction. The structural evolution during charge–discharge follows an O3–O1'–O1 sequence (Figure 4e), consistent with oxygen‐redox‐driven layered oxides such as Na_2_IrO_3_. The incorporation of the 5*d* cation Ir introduces strong Ir–O covalency, which further stabilizes the anionic redox process by mitigating oxygen loss [173].

Beyond, Na_2_MnO_3_, Ti‐based Na‐rich cathode has also been studied for its anionic redox activity [174]. The Na_2_TiO_3_ composition synthesized by high‐energy ball‐milling showed a reversible capacity of 217 mAh g^−1^. Further, the electrochemical performances of the cathode are improved (336 mAh g^−1^) by doping Cr and carbon coating. In addition, Na‐excess Na_2_TiO_3_‐NaMnO_2_ cathode demonstrated a reversible capacity of 200 mAh g^−1^ corresponding to Mn^3+^/Mn^4+^ redox activity [175]. Very recently, F‐substituted Mn‐based Na‐rich Na_1.2_Mn_0.8_O_1.5_F_0.5_ has been demonstrated as a cathode for SIBs. The material showed improved anionic redox properties of high capacity, capacity retention, and stable cycling stability up to 300 cycles owing to the distortions and reduction in TM valency [176].

#### 4.1.2 4*d* Transition Metal‐Based Layered Oxides

Na‐rich layered oxides such as Na_2_MO_3_ and Na_3_MO_4_ with 4*d* TMs are considered high‐performance anionic redox cathodes. In particular, Ru‐based Na‐rich layered oxides (M = Ru) serve as ideal model systems for anionic redox, as Ru can adopt multiple stable oxidation states (Ru^5+^, Ru^6+^, Ru^7+^) in distinct coordination environments, including octahedral, trigonal bipyramidal, and tetrahedral ones. The polymorphism of Ru compounds, together with Na/TM ordering, allows systematic tuning and observation of oxygen redox behavior. Moreover, the 4*d* orbitals of Ru promote strong TM–O hybridization, and the local Ru–O environment, along with chemical pressure from alkali ions, affects O 2*p* states, enabling reversible oxygen participation in charge compensation. These characteristics make Ru particularly well‐suited for studying the mechanisms of anionic redox in Na‐rich layered oxides [123, 136].

**FIGURE 5:**
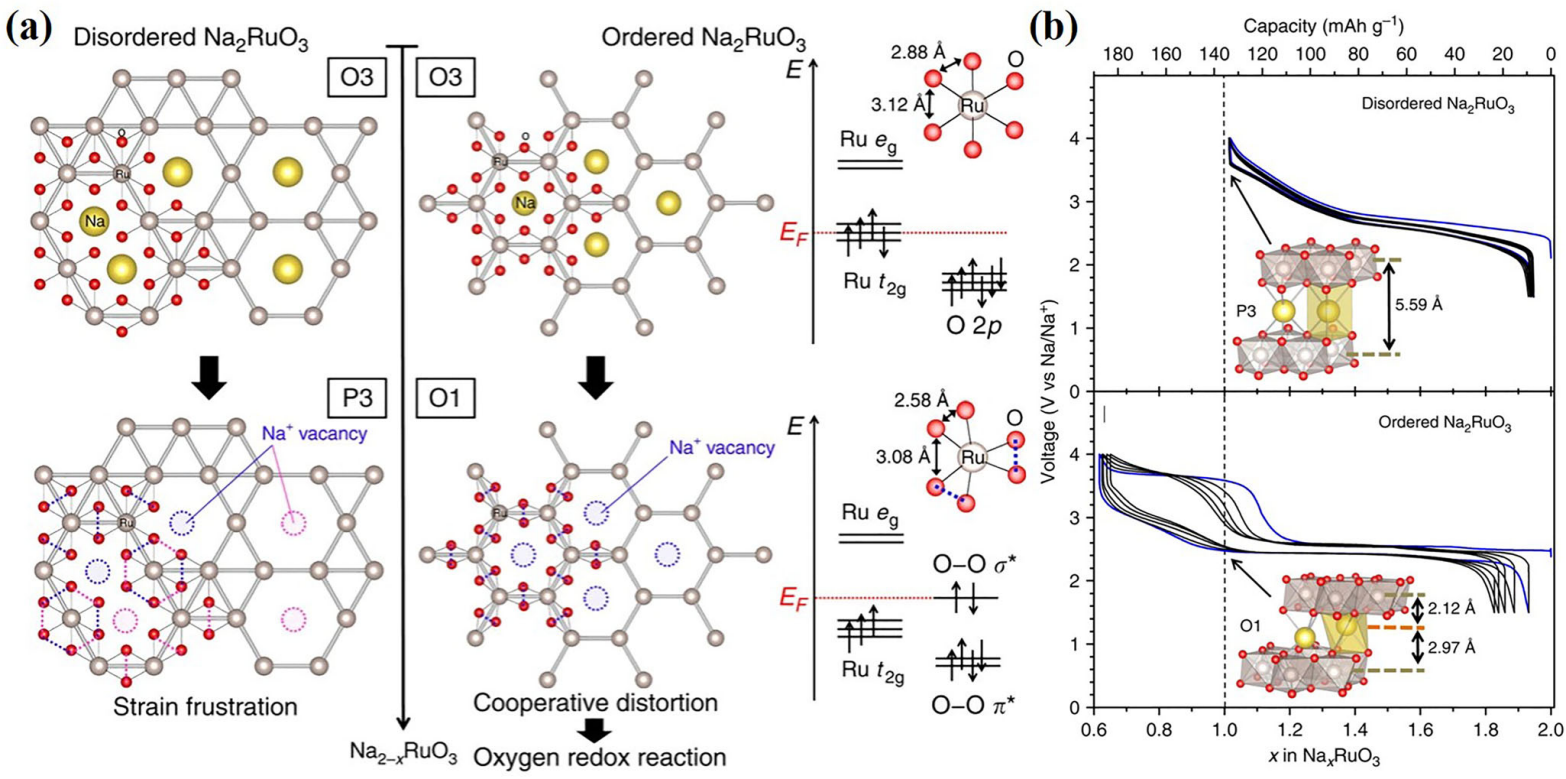
(a) Schematic representation of the structural changes for disordered Na_2_RuO_3_ and ordered Na_2_RuO_3_. Disordered Na_1_RuO_3_ cannot accommodate the RuO_6_ distortion due to strain frustration, which prevents the oxygen redox reaction. Ordered Na_2_RuO_3_ can distort cooperatively to raise the energy level of the antibonding σ^*^ orbital of the O–O bond, leading to the oxygen redox reaction [177]. Reprinted with permission [177] CC‐BY‐4.0. (b) Galvanostatic cycling curves recorded at 30 mA g^−1^ for disordered and ordered Na_2_RuO_3,_ with the first cycle highlighted in blue. Insets show the coordination environment of Na at *x* = 1.0 for each phase. (Reprinted with permission. Copyright 2016 Nature Publishing Group).

Even though Na_2_RuO_3_ is isostructural with Li_2_RuO_3,_ which follow different charge storage mechanism [177], Na‐rich Na_2_RuO_3_ is a well‐known anionic redox‐active material that adopts a O3‐type (*R‐3m* space group) structure. Among them, one polymorph has honeycomb‐ordered [Na_1/3_Ru_2/3_]O_2_ slabs, while the other possesses disordered [Na_1/3_Ru_2/3_]O_2_ slabs (Figure 5a) [177]. The honeycomb‐ordered Na_2_RuO_3_ exhibits a high reversible capacity of approximately 180 mAh g^−1^, which corresponds to the extraction of around 1.3 Na^+^ ions per formula unit, exceeding the theoretical capacity expected from the Ru^4+^/Ru^5+^ cationic redox alone (1 electron transfer). In contrast, the disordered form of Na_2_RuO_3_ delivers a lower capacity of about 135 mAh g^−1^, which aligns with the redox activity of Ru involving a single electron. As illustrated in Figure 5b, the shortened O–O bond length of 2.580(4) Å observed in the distorted RuO_6_ octahedra facilitates orbital rearrangement. Specifically, it causes the energy level of the oxygen–oxygen antibonding σ^*^ orbital to shift closer to the Fermi level, thereby enabling additional charge compensation via anionic redox processes in Na_2‐_ *_x_* RuO_3_.

A recent theoretical report shows that the aliovalent doping (Al^3+^, Sn^4+^, Mg^2+,^ and Na^+^) on Na_2_RuO_3_ can tune the anionic redox reactions, and particularly the Al^3+^ doped Na_2_Ru_0.5_Al_0.5_O_3_ exhibited enhanced stability and reversibility of anionic redox by altering TMO layer [178]. Recently, yttrium‐doped Na‐rich Na_2_ZrO_3_was found to exhibit a large reversible capacity of ∼180 mAh g^−1^ with excellent cycling stability [179]. Also, the effect of Co doping in Na_2_RuO_3_ was studied, where Co acted as a redox mediator and the optimal Co‐doped cathode Na_2_Ru_1.9_Co_0.1_O_3_offering a large reversible capacity of 177 mAh g^−1^ [180].

In addition to Na_2_RuO_3_, another type of Na‐rich cathode Na_3_RuO_4_ (Na[Na_1/2_Ru_1/2_]O_2_) with Ru^5+^, has also been demonstrated as both cationic and anionic redox active cathode for SIBs [136, 181]. The high ratio of Na/Ru of this composition leads to an increase in initial charge capacity to 321 mAh g^−1^that corresponds to removal of 2.7 Na per formula unit. The in situ Raman studies revealed the formation of peroxo‐species and Na_2_O during cycling.

#### 4.1.3 5*d* Transition Metal‐Based Layered Oxides

**FIGURE 6:**
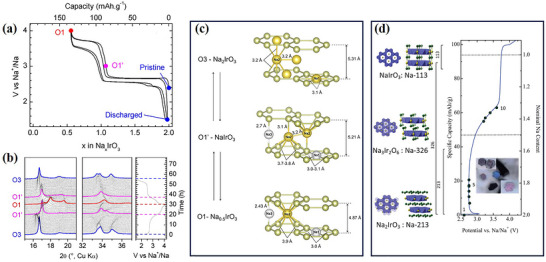
(a) Galvanostatic charge‐discharge profile (first 2 cycles) and (b) In situ XRD studies of Na_2_IrO_3_ showing phase changes of O3–O1′–O1. (c) Possible Na positions for the O3, O1′ and O1‐type structures [182]. Reprinted with permission [182] 2016, American Chemical Society (d) Galvanostatic charge profile of single crystal Na_2_IrO_3_ along with its crystal structural changes [183]. Reprinted with permission [183]. 2019 WILEY‐VCH Verlag GmbH & Co. KGaA, Weinheim.

Na‐rich layered oxides with 5*d* transition metals (Na_2_IrO_3_) are attractive owing to their stable anionic redox behavior. Theoretical studies demonstrated that the increase in M–O covalency for 5*d* (Ir) TMs relative to 3*d* (Mn) and 4*d* (Ru) TMs leads to improved anionic redox reactions and reduced oxygen release. For instance, Na_2_IrO_3_ delivered a high capacity of 130 mAh g^−1^ corresponding to 1.5 Na^+^ transfer per formula unit and low oxygen loss as shown Figure 6a. In situ XRD results and the corresponding structural modifications revealed that the charge storage mechanisms in Na_2_IrO_3_ follow comparable structural changes of O3–O1′–O1 as discussed in the Na_2_RuO_3_(Figure 6b,c). The strong covalency between Ir and O suppresses cation migration and oxygen evolution due to the strong overlap of the Ir 5*d* band and O2*p* bands [182]. The Na_2_IrO_3_ delivers a capacity of 130 mAh g^−1^ similar to the Li‐analogue Li_2_IrO_3_ due to the cationic redox reaction Ir^5+^/Ir^6+^ and anionic redox. Highly ordered single crystal Na_2_IrO_3_ was also investigated [183]. The phase transitions and electrochemical results of the single crystal Na_2_IrO_3_ depicted that only cationic redox activity Ir^4+^/Ir^5+^ is possible during charge with no evidence for anionic redox activity(Figure 6d). Further, Li substitution in Na_2_IrO_3_ was also investigated. Na_1.5_Li_0.5_IrO_3_ (Na[Li_1/3_Ir_2/3_]O_3_) shows honeycomb like structure with Li‐ions situated at the center of the Ir honeycomb [184]. Se‐based Na‐rich Na_2_SeO_3_ with the space group of *P*2_1_/*c* was also proposed as high capacity cathode (232 mAh g^−1^) due to its anionic redox by theoretical calculations [185]. However, the cycling stability of the cathode was poor owing to the selenium dissolution and structural instability problems.

### 4.2 O3‐Types Layered Oxide Cathodes

In the realm of battery technology, particularly in the context of SIBs, different types of layered oxides serve as cathode materials. These include P2, P3, O2and O3‐type layered oxides [27, 43, 138, 186]. Among them, O3‐type layered oxides have emerged as promising candidates for cathode materials due to certain advantages, particularly in full‐cell applications. While O3‐type layered oxides offer advantages in terms of sodium content and performance in full‐cell applications, they are also subject to multiple phase transitions (O3−O′3−P3−P′3−P3′− O1), especially at high voltages. Indeed, exposure to air can induce structural changes in O3‐type layered oxides, converting to O′3 and P3 phases. Addressing these challenges associated with complex phase transitions is crucial for improving the performance and longevity of SIBs utilizing O3‐type layered oxides as cathode materials [156, 187, 188, 189, 190, 191, 192, 193].

**FIGURE 7:**
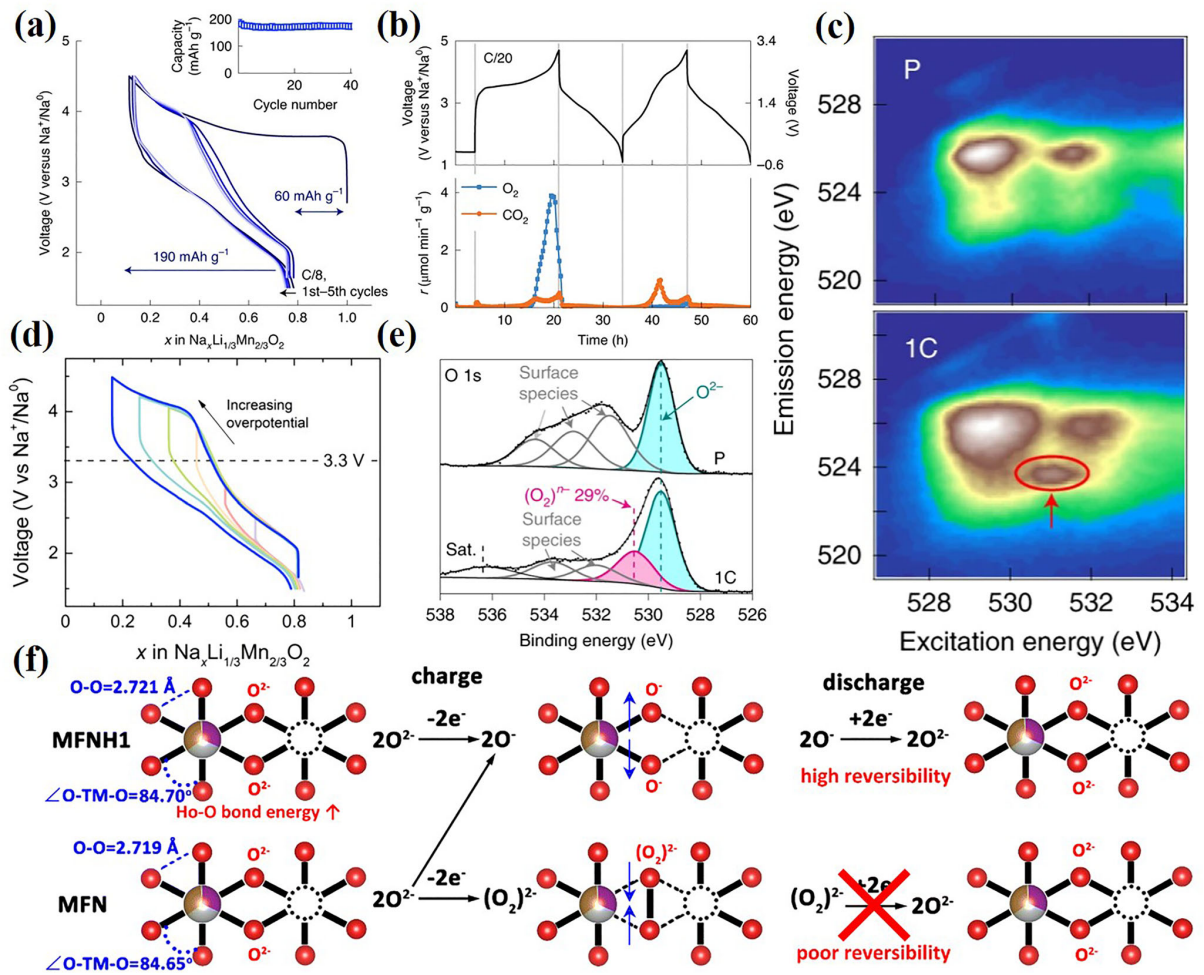
(a)The voltage profile of the initial five galvanostatic cycles of NaLi_1/3_Mn_2/3_O_2_ versus metallic sodium is depicted at a C/8 rate (1C = 285 mAh g^−1^) within the range of 1.5 to 4.5 V. The inset illustrates the capacity retention across 40 cycles. (b) During the initial and subsequent cycles, OEMS gas analysis was conducted on NaLi_1/3_Mn_2/3_O_2_ cycled against Na_3_V_2_(PO_4_)_3_ within the voltage range of −0.5 to 3.1 V (right axis), which corresponds to 1.2–4.7 V versus Na^+^/Na^0^ (left axis) after conversion. (c) Effect of voltage window on polarization in the second cycle while increasing the upper cutoff voltage. (d) Ex situ HAXPES (with photon energy hυ =  6900 eV) of O1*s*. (e) Ex situ O K‐edge mRIXS spectra were obtained for both pristine (P) and samples charged to 4.5 V at a rate of 1 C. A red arrow and circle highlight the oxidized oxygen feature at a fully charged state, observed at 531.0 eV excitation energy and 523.7 eV emission energy. Additionally, a satellite peak labelled “Sat.” is observed, consistent with previous observations [191]. Reprinted with permission [191] 2021, Springer Nature Limited, All rights reserved. (f) Schematic diagram of the oxygen oxidation reaction process in MFNH1 and MFN materials [192]. Reprinted with permission [192] 2023, Royal society of chemistry.

Wang et al. reported O3‐type NaLi_1/3_Mn_2/3_O_2_ cathode synthesized via a ceramic method exhibiting anionic redox at high voltage with a capacity of ∼190 mAh g^−^^1^.The subsequent discharge showed a persistent S‐shaped curve (Figure 7a), indicating an electrochemically induced structural transformation during the initial charge. During the initial charge, 0.9 Na^+^ ions were extracted, but only 0.67 Na^+^ ions were reinserted during discharge, resulting in a sustained reversible capacity. OEMS data showed less than 1% O_2_ release during the subsequent charge (Figure 7b). The upper cut‐off voltage was progressively increased (Figure 7c) to highlight the development of voltage hysteresis at high‐voltage redox, which is an important consideration for understanding polarization in Na‐rich cathodes. While the charge profiles remain similar, the discharge curves drop at >3.3 V with a rapid rise in overpotential, indicating that hysteresis primarily develops during the latter stage of charge. O1*s* HAXPES (Figure 7d) and mRIXS (Figure 7e) spectra revealed an extra peak at 530.5 eV correlated to previous reports, indicating the trace of oxidized lattice oxygen (O^n−^, where n < 2), suggesting that the oxygen atoms have lost electrons and are in an oxidized state [191].

The following passage discusses the importance of Oxygen Anion Redox (OAR) in the performance and stability of cathodes. It highlights challenges associated with initiating and sustaining OAR and proposes a strategy to enhance OAR activity. Doping with Ho (holmium) in the O3‐NaMn_1/3_Fe_1/3_Ni_1/3_O_2_ cathode (MFN and MFNH1) involves the modification of Na/Mn anti‐site. Additionally, oxygen evolution is mitigated due to the pinning effect of Mn^2+^ and the formation of Ho–O bonds. As a result, it perks up the capacity from 146.8 to 184.9 mA h g^−^^1^. Moreover, there is a notable enhancement in capacity retention, rising from 40.3% to 90.0% [192].

Voronina et al. reported O3‐Na[Ni_2/3_Ru_1/3_]O_2_ compound displaying a notable capacity above ∼154 mAh g^−1^. This high capacity and stable cycling performance are attributed to the sequence of 3*d* (Ni) and 4*d* (Ru) TM within the structure, which promotes high covalence in the material [194]. Guo et al. integrated boron into the O3‐NaLi_1/9_Ni_2/9_Fe_2/9_Mn_4/9_O_2_ to prevent the formation of additional plateaus resulting from anionic redox (>4.0 V vs. Na^+^/Na). Boron incorporation formed B–O covalent bonds, enhancing structural integrity and suppressing oxidation. Consequently, this cathode achieved a capacity of 160.5 mAh g^−1^ (at 25 mA g^−1^) retaining ∼82.8% after 200 cycles (at 250 mA g^−1^). DEMS and DFT calculations were exploited to investigate the influence of boron incorporation [195].

A solid‐state synthesis using varied sintering atmospheres produced stoichiometric NLCR‐Ar and non‐stoichiometric NLCR‐Air with oxygen vacancies. NLCR‐Ar had a higher initial capacity (203.2 mAh g^−1^) than NLCR‐Air 175.1 mAh g^−1^ (0.1C). However, NLCR‐Air showed better capacity retention, maintaining 87.7% after 1000 cycles (20C), compared to NLCR‐Ar (66.3% after 700 cycles). Research showed that sXAS effectively reveals the overall oxygen redox behavior during electrochemical cycling. The Oxygen vacancies in NLCR‐Air mitigated over‐oxidation, reducing irreversible O redox processes. Theoretical analysis indicated the absence of oxygen vacancies in NLCR‐Ar hinders Na kinetics, highlighting their importance in efficient charge transfer [196].

Yttrium substitution in NaNi_1/3_Fe_1/3_Mn_1/3_O_2_ (NFM) to form NaNi_1/3_Fe_1/3−0.01_Mn_1/3_Y_0.01_O_2_ (NMFY1) significantly enhanced the electrochemical performance. The robust Y–O bond stabilizes the crystal structure, prevents TM layer slipping and inhibits irreversible phase transitions, enhancing cycling performance. Furthermore, SIBs layer is expanded, increasing the diffusion coefficient and rate capability while reducing the manganese oxidation (Mn^3+^/Mn^4+^) ratio so as to mitigate the Jahn–Teller effect [197].

Zhu et al. proposed a strategy involving double (Li) and (B) doping with in NaNi_0.3_Fe_0.3_Mn_0.4_O_2_. This approach effectively suppresses irreversible phase transitions and the dissolution of transition metals. Notably, the material demonstrates excellent performance, achieving a capacity around (175 mAh g^−1^ at 0.2C) and retaining 87.3% capacity upon 100 cycles (1C) [198]. This study presents an O3‐Na_0.8_Li_0.2_Fe_0.2_Ru_0.6_O_2_ designed for a stable 4.5 V SIB. The dual‐reductive coupling mechanism in this cathode suppresses the O3−P3 phase transition and enhances oxygen redox reversibility at high voltages. In/ex situ XRD results revealed that P3 phase transition is inhibited during cycling, replaced by O1′ and O1 phases. The reversible migration of Li ions plays a “pinning” role, preventing the slabs from gliding from octahedral to prismatic positions, thereby improving the structural robustness. XANES and XPS analyses demonstrated the Fe/Ru dual‐reductive coupling mechanism activates during deep desodiation, enhancing anionic redox reversibility through the formation of strong Fe/Ru−(O−O) covalent bonds [199].

**FIGURE 8:**
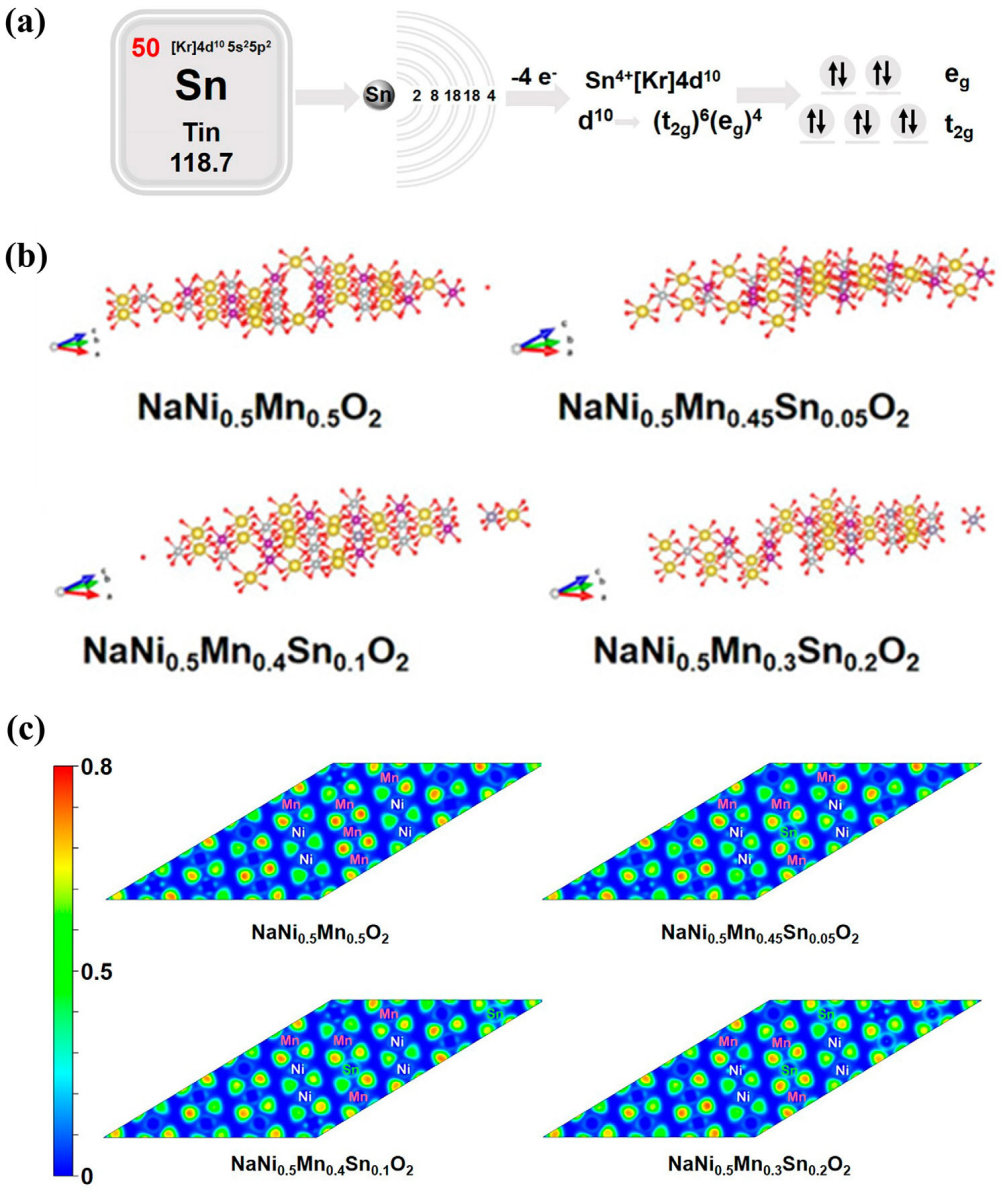
(a) Schematic illustration of the outer electron configuration (OEC) of Sn^4+^. (b) Optimized crystal structures of NaNi_0.5_Mn_0.5‐_ *_x_* Sn*_x_* O_2_ electrode materials. (c) A detailed analysis of the ELF (Electron Localization Function) of the valence electrons along the [001] direction [200]. Reprinted with permission [200] 2023, American Chemical Society. All rights reserved.

As shown in Figure 8a, Sn belongs to group IVA of the fifth period, and its electronic configuration follows the Aufbau principle and the Pauli exclusion principle. Upon losing four valence electrons, Sn^4+^ adopts a closed‐shell electronic configuration ([Kr]4*d* ^10^]. Owing to the fully filled *d* orbitals, Sn^4+^ contains no unpaired electrons, and crystal‐field splitting does not introduce partially occupied *e_g_* or *t_2g_* states. As a result, Sn^4+^ is electronically inactive and does not participate in redox processes.

To further examine the local electronic structure, first‐principles calculations were carried out for NaNi_0.5_Mn_0.5‐_ *_x_* Sn*_x_* O_2_, and the optimized structural models are shown in Figure 8b. Consistent with density‐of‐states analysis, the states near the Fermi level are mainly derived from O‐2*p* orbitals hybridized with Ni/Mn *d* states, supporting anionic redox reaction behavior. Electron Localization Function (ELF) analysis reveals that the oxygen coordinated with Ni and Mn in NaNi_0.5_Mn_0.5_Sn_x_O_2_ exhibits a lower ELF value compared to the undoped NaNi_0.5_Mn_0.5_O_2,_ indicating stronger Ni/Mn–O interactions.

Owing to its closed‐shell electronic configuration, Sn^4+^ does not participate in TM–O hybridization and remains electronically inactive. This suppresses charge delocalization within the transition‐metal layers and contributes to an elevated redox potential (Figure 8c). Notably, among the studied compositions, moderate Sn substitution results in the strongest Ni/Mn–O interaction, supporting the role of Sn in stabilizing anionic redox [200].

In this study, Li–Mg–Ca–Sb co‐doping of O3‐Na_0.8_Ni_0.4_Fe_0.2_Mn_0.4_O_2,_ where partial substitution with Sb^5+^, Li^+^, Mg^2+^ and Ca^2+^ yields Na_0.8_Ni_0.3_Fe_0.2_Mn_0.3_Li_0.1_Mg_0.02_Ca_0.05_Sb_0.03_O_2_ (LMCS NFM). The co‐doped material shows suppressed phase transitions above 4.1 V and reduced anisotropic lattice strain, which helps maintain particle stability compared with the undoped counterpart. In addition, the authors associate the improved electrochemical reversibility with a more stabilized oxygen redox process, attributed to the presence of Sb^5+^ and alkali/alkaline‐earth dopants (Li^+^, Mg^2+^ and Ca^2^) in the lattice. Together, these results indicate that co‐doping provides a practical means to balance high‐voltage operation and structural robustness in O3‐type layered sodium cathodes [201].

Lin et al. performed DFT calculations for the redox reaction of O3‐type Na_0.99_K_0.01_Ni_0.5_Mn_0.4_Ti_0.1_O_2_, showing that Ni 3*d* and O 2*p* orbitals dominate close to the Fermi level, indicating that Ni ion redox reactions facilitate electron transfer. The conduction band energy levels of the TM 3*d* and O 2*p* orbitals are shifted higher in the doped material, indicating a higher discharge voltage. This co‐substituted cathode exhibits a high‐rate capacity of 97 mAh g^−1^(at 5 C) [202].

Dong et al. utilized different characterizations (*operando* X‐ray diffraction, XAS, high‐resolution neutron powder diffraction, and neutron pair distribution functions, DFT, and neutron pair distribution function (nPDF)) to investigate both charge compensation mechanism and phase evolution (>4.1 V) of O3‐NaFe_0.5_Mn_0.5_O_2_. The study suggests Fe migration into the Na layer as Na^+^ depletes, affecting structural integrity [203]. This study illustrates how transition metal (TM) distributions affect doping in O3‐Na*_x_* (Ni_y_Sb_1‐y_)O_2_ (ordered/disordered) by introducing Cu. Theoretical calculations show that TM disorder promotes antibonding orbital formation between O 2*p* and Cu 3*d*, hindering Cu migration and enhancing Na diffusion kinetics. Thus, Cu migration occurs in the honeycomb‐ordered phase and not in the case of the disordered phase [204].

In O3‐NFM (NaNi_1/3_Fe_1/3_Mn_1/3_O) layered cathodes, cycling at 4.0 V initiates surface degradation such as cracking, corrosion, and phase transitions, progressing into the grain interior and causing capacity decay. At 4.3 V, accelerated surface degradation and significant bulk failures occur, including intragranular cracking and nanovoid formation due to vacancy clustering and transition metal condensation. Thus, stability of the cathode surface/interface is enhanced at low‐voltage, while preserving bulk structure stability is crucial for high‐voltage cycling [205].

Zhang et al. developed a Li/Sn co‐substituted O3‐Na_0.95_Li_0.07_Sn_0.01_Ni_0.22_Fe_0.2_Mn_0.5_O_2_ (LSNFM) cathode via the solid‐state method. The elements like Mn, Ni, and Fe attributed to the improvement of electronic structures and also not only reducing Mn^3+/4+^ activity, but also increasing Ni^2+/3+^ and Fe^3+/4+^activity (2.0–4.0 V). Jahn–Teller effect of Mn^3+^ is supressed below 2.5 V and enhances TM ion redox reactions above 2.5 V [206]. The incorporation of Li in the TM (O3‐Na[Li_0.05_(Ni_0.25_Fe_0.25_Mn_0.5_)_0.95_]) showed a sharp diffraction peak. The presence of strong Li−O bonds compared to Ni−O and Mn−O bonds enhances the electrochemical properties and structural integrity. This strong bond can either trigger or suppress the anionic redox process [207]. Doping with Ti forms a Ti−O bond (662 kJ/mol) with a higher bond energy than Ni−O (391.6 kJ/mol), Fe−O (409 kJ/mol), and Mn−O (402 kJ/mol) bonds. This leads to a reduction in the TM−O bond length in the Ti‐doped cathode, thereby improving structural stability and reversibility of anionic redox reactions by making oxygen stripping more difficult [208].

### 4.3 Na‐Deficient Layered Oxides

Sodium‐based layered metal oxides form the most practical cathodes for sodium‐ion batteries (SIBs). Compared to O3‐type materials, Na‐deficient P2‐type layered oxides show better structural integrity and kinetics due to their direct prismatic diffusion pathways. Additionally, these cathode materials exhibit reversible oxygen redox reactions, wherein oxygen ions participate in charge compensation during battery cycling. This anionic redox chemistry not only contributes to higher energy densities but also offers insights into designing more efficient and stable sodium‐ion battery systems.

However, critical disadvantages such as low sodium content, irreversible phase transformation, transition metal cation migration, and loss of oxygen during Na extraction/insertion that cause poor reversible capacity and cycle stability at high voltages limit their practical deployment. While TM site substitution in layered oxide cathodes have been widely studied, there are relatively few reports on the anionic redox activity. The active involvement of oxygen redox couple could open up a pathway for the high‐energy‐density SIBs. Anionic redox activity in P2‐type layered oxide materials have been reported for sodium‐ion battery applications [209, 210, 211].

**FIGURE 9:**
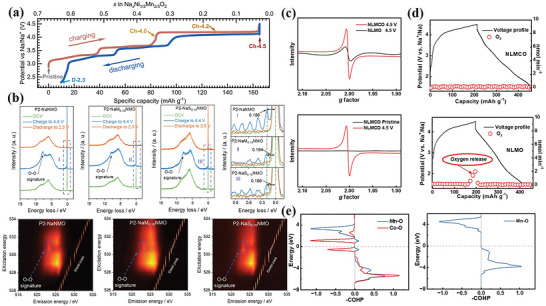
(a)The charging and discharging profiles of Na_2/3_Ni_1/3_Mn_2/3_O_2_ at 0.1C (1C = 160 mA g^−1^) [218]. Reprinted with permission [218] 2020 Elsevier Ltd. All rights are reserved. (b) O K‐edge RIXS spectra P2‐NaNMO, P2‐NaM_0.10_ NMO, and P2‐NaS_0.10_NMO at OCV, charged to 4.4 V, and discharged to 2.5 V states. Magnified RIXS spectra for each sample (I, II, III) in the three states [220]. Reprinted with permission [220] 2024 WILEY‐VCH Verlag GmbH & Co. KGaA, Weinheim.(c) EPR spectra of fully charged NLMCO and NLMO electrodes under the same measurement conditions. EPR spectra of pristine and fully charged NLMCO electrodes. (d) In situ DEMS spectra collected during the initial charge and discharge of NLMCO and NLMO electrodes. (e) COHP profiles of Mn–O and Co–O bonds for NLMCO, Mn–O bonds for NLMO [222]. Reprinted with permission [222] 2023 WILEY‐VCH Verlag GmbH & Co. KGaA, Weinheim.

Among the Na*_x_* TMO_2_ family, Na‐based layered Ni‐Mn system Na_0.67_Ni_0.33_Mn_0.67_O_2_ (NNMO) have received wide attention on account of the two‐electron transfer and high redox potential of the Ni^2+^ ions leading toa practical capacity of 173 mAh g^−1^ [212, 213, 214, 215]. Furthermore, the Na‐deficient NNMO has garnered great interest due to its air stability, phase stability, and highly reversible electrochemical profile with well‐defined plateaus upon cycling along with a low voltage hysteresis of about 0.1 V (Figure 9a). Risthaus et al. reported the NNMO with a prominent discharge capacity of 228 mAh g^−1^ in the voltage range of 4.5‐1.5 V. The overall capacity was contributed by the cationic and anionic activities of Ni, Mn (surface) and oxygen ions. It was concluded from XAS analysis that anionic redox activity happens at higher voltages [216, 217].

Later Dai et al. used resonant inelastic X‐ray scattering (RIXS) characterization to explain the involvement of anionic redox along with cationic redox of Ni^2+/4+^ in the NNMO system. It was evidenced by Ni–L Total Electron Yield (TEY) and Total Fluorescence Yield (TFY) spectra that upon charging to 4 V, 80% of Ni^2+^ to Ni^3+^ oxidation took place. Anionic redox occurs while charging from 4‐4.5 V, while Mn retains the +4‐oxidation state [218, 219]. Although anionic redox in the higher voltage contribute toward the storage capacity in P2‐type layered oxide cathodes, capacity degradation is expected due to the transition metal cation migration and loss of oxygen. It can be mitigated by substituting inactive elements such as Mg^2+^ and Li^+^ into the transition metal sites.

Li et al. reported the impact of magnesium and scandium doping in the Ni^2+^ lattice site of P2 type Na_0.67_Ni_0.33_Mn_0.67_O_2_ system. By using RIXS analysis, the consequence of Mg^2+^ and Sc^3+^ doping on anionic activity was investigated [220]. Figure 9b displays the RIXS values (O K‐edge) for the pristine P2‐type cathodes Na_0.67_Ni_0.33_Mn_0.67_O_2_, Mg‐doped NaM_0.10_NMO, and Sc doped NaS_0.10_NMO during initial cycle between 4.4 V to 2.5 V and at open circuit voltage condition. A peak (8 eV) corresponding to the formation of typical oxygen‐oxygen bond was observed in the charged condition.

Similarly, several compositions with varying Ni/Mn rations also exhibit anionic redox. In the work by Thanwisai et al., Mg was introduced into the P2‐type Na_0.62_Ni_0.25_Mn_0.75_O_2_ cathode to effectively regulate anionic redox activity, thereby enhancing electrochemical stability. Doping Mg (10 mol%) mitigated the P2–O2 phase transition and minimized irreversible anionic redox tested by electrochemical and material characterization. The optimized sample displayed a capacity retention of 92% (half‐cell) and 95% (full cell) after 100 cycles and 170 cycles, respectively [221].

Jin et al. reported a novel P2‐Na_0.75_Ca_0.04_[Li_0.1_Ni_0.2_Mn_0.67_]O_2_ cathode displaying Mn and O synergetic redox mechanism. The capacity loss due to anionic release was complemented by the increased Mn^3+/^Mn^4+^ by the limited release of oxygen. As a result, significant capacity retention of 95.2% was observed over 200 cycles at the rate of 5C. Due to superior Na^+^ diffusion in P2frameworks, the half‐cell displayed an excellent rate capability of 133.1 mAh g–^1^ (0.1C) and 68.8 mAh g–^1^ (20C), and the full‐cell exhibited good rate capability (57.6 mAh g–^1^ at 60 C) and energy density (≈ 271.3 Wh kg–^1^).

**FIGURE 10:**
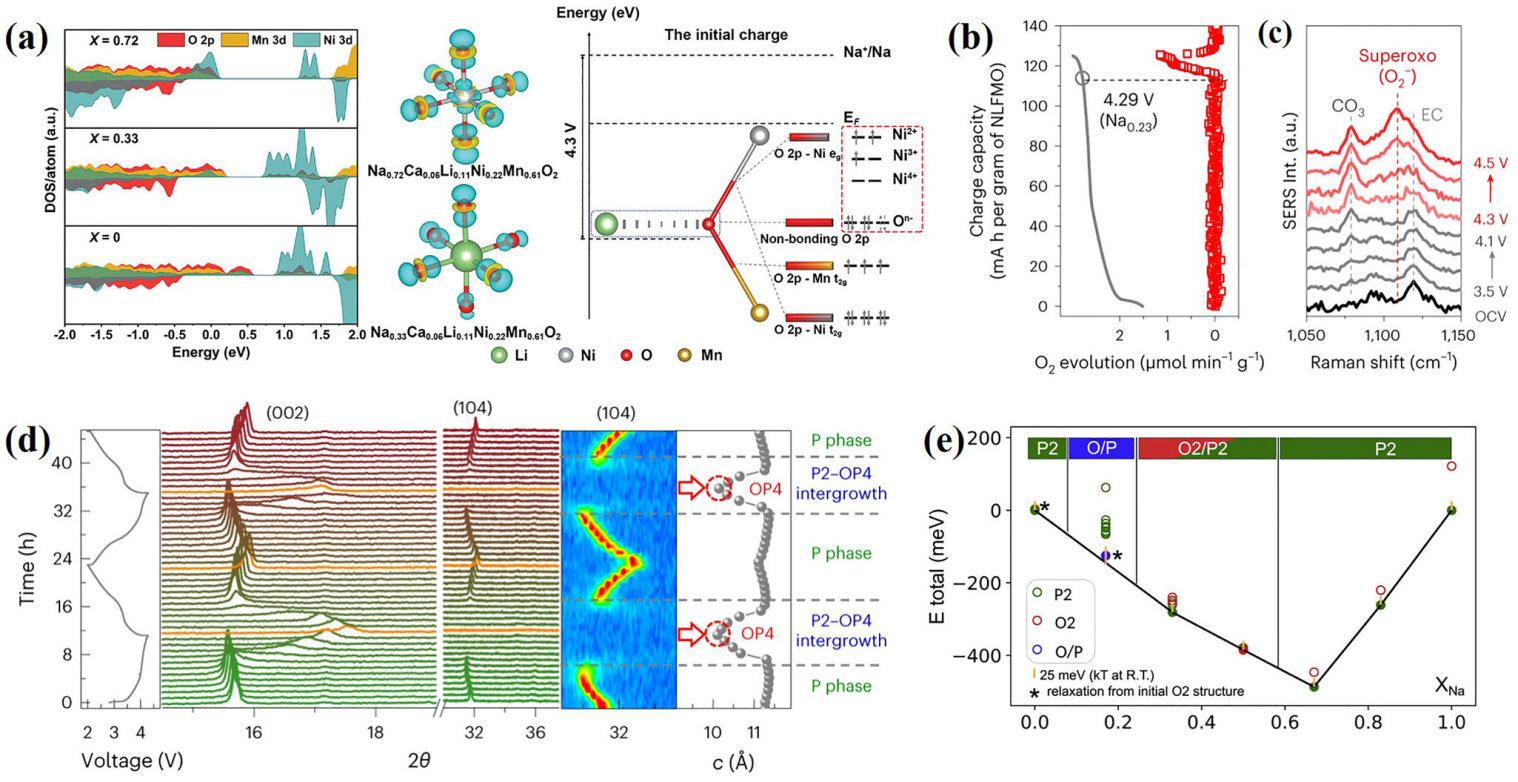
(a) DFT computations. Projected DOS of the O 2*p*, Mn 3*d*, and Ni 3*d* orbitals of the layered oxides (Na_x_Ca_0.06_[Li_0.11_Ni_0.22_Mn_0.61_]O_2_) with varying Na contents [223]. Reprinted with permission [223] 2022 WILEY‐VCH Verlag GmbH & Co. KGaA, Weinheim. (b) The evolution rate of gaseous O_2_ harvested by OEMS during the initial charging process. To present the capacity dependence, the initial galvanostatic charge curve is shown for clarity (grey trace). (c) Potential‐dependent in situ SERS results collected for the NLFMO cathode upon initial charging. (d) In situ XRD patterns of the NLFMO cathode obtained during the initial two cycles (2.0–4.3 V cycling) [227]. Reprinted with permission [227] 2024 springer nature limited. (e) Phase stability diagram for both the phases (P2 and O3) with respect to the biphasic reaction (experimental) with respect to varying Na composition [228]. Reprinted with permission [228] 2022 Elsevier Ltd. All rights are reserved.

Density Functional Theory (DFT) calculations have been employed to investigate charge compensation mechanism involved during cycling. Figure 10a displays the density of states with respect to varying Na concentrations [223].

In summary, Ni redox prevailed the overall charge compensation process during sodium extraction because of the depleted electron concentration at Ni 3*d* orbital over the Fermi level. With increased extraction of Na*_x_*, electron density of oxygen 2*p* dominated over the Ni 2*p* orbitals which confirmed the predominant contribution of oxygen toward the achieved capacity at profound desodiation. Similarly, Shen et al. unveiled a novel P2‐Na_0.80_Li_0.08_Ni_0.22_Mn_0.67_O_2,_ which displayed the Mn activation mechanism by triggering the partial release of oxygen with cycling upto 4.3 V. This contributed to the increased activation of manganese redox, resulting in retaining the initial capacity. The as‐prepared cathode displayed an excellent discharge capacity of 134.8 mAh g^−1^ at 0.1 C and a retention of capacity up to 85.2% after 1000 cycles at 10 C. A fully solid‐solution pathway with lesser volume change of 1.04% upon cycling was revealed by the in situ X‐ray diffraction analysis [224].

Recently, Lu et al. developed a P2 type Na_0.7_[Li_0.2_Mn_0.7_Co_0.1_]O_2_ (NLMCO) by reversing the asymmetric lattice oxygen activity. In comparison to Na_0.75_[Li_0.25_Mn_0.75_]O_2_ (NLMO), NLMCO exhibited a higher energy density (729.7 Wh kg^−1^). The Co introduced into the TM sites enhanced the TM–O bonding, thereby improving the Na diffusion kinetics and reducing energy band gap. Additionally, it enables the reversible creation and elimination of electron holes within lattice upon cycling [222].

In the EPR spectra (Figure 9c), the fully charged (4.5 V) states of NLMCO and NLMO exhibited the notable peaks at g = 2.004 when compared to pristine cathode indicating the presence of unpaired electron as a consequence of oxygen redox activity. The Co doped NLMCO displayed a higher intensity peak in contrast to the NLMO at the 4.5 V state, pointing out increased oxygen activity which is highly reversible in NLMCO. The evolution of oxygen in both samples during cycling was characterized by in situ DEMS analysis. Oxygen release was not detected in Co doped sample electrode compared to pristine when charged upto 4.2 V (Figure 9d). To analyse transition metal‐oxygen interactions, crystal orbital Hamilton population (COHP) was further orchestrated.

According to the COHP analysis, bonding interaction is indicated by a negative sign and antibonding interaction is expressed by a positive value. A significant improvement in bonding interaction between transition metals and oxygen is observed underneath the Fermi level in the Co‐doped NLMCO when compared to the pristine sample. Strong TM–O interaction is apparent in NLCMO upon desodiation as a result of anionic oxygen stabilization (Figure 9e).

Substitution of Cu in P2‐type layered oxides has emerged as an effective strategy to modulate anionic redox and enhance cathode stability. Hu et al. demonstrated that controlled Cu incorporation in Na_0.73_Li_0.21_Mn_0.74_Cu_0.05_O_2_ (NLMCO) stabilizes the Mn–O framework while suppressing irreversible oxygen redox, as demonstrated by combined sXAS and EPR studies.

In the Cu‐doped material, the characteristic EPR signal associated with trapped molecular O_2_ in fully charged pristine NLMO is absent, confirming mitigation of deleterious oxygen activity. Cu substitution also inhibits Mn^3+^ formation, stabilizes Li^+^ intercalation/extraction, and mitigates local structural distortions linked to oxygen redox, leading to enhanced cycling stability. As a result, NLMCO delivers a reversible capacity of 150.3 mAh g^−1^ at 0.5 C with 84.9 % retention over 60 cycles. The filled *d* ^8^ configuration of Cu^2+^ strengthens the Cu–O–Mn bonding network, localizes electrons around Cu^3+^, and attenuates oxygen redox, providing a robust approach to stabilize the transition‐metal environment and achieve high‐stability cathodes for sodium‐ion and lithium‐ion batteries [225].

In contrast, Fe‐Mn‐based P2 cathodes, such as P2‐ Na_2/3_Fe_0.5_Mn_0.5_O_2_, primarily operate through Fe^3+^/Fe^4+^cationic redox. In these sodium systems, anionic redox is less pronounced due to the lower Fe–O covalency compared with lithium analogs, allowing Fe^3+^ oxidation to dominate while minimizing lattice oxygen participation. Fe^4+^/Fe^3+^redox dominated P2‐Na_2/3_Fe_0.5_Mn_0.5_O_2_ delivered a charging capacity of 190 m Ah g^−1^ as reported by Yabuuchi et al. [226]. This distinction highlights the differing roles of cationic and anionic redox in stabilizing P2 layered oxides and underscores the need to tailor dopants or substitutions depending on the transition‐metal chemistry.

Environmentally benign and inexpensive P2‐type Fe‐Mn based compositions has attracted considerable attention as cathodes for sodium‐ion batteries. Nevertheless, their practical application is limited by poor air stability and irreversible structural transformations.

Recently, Lee et al. reported redox inactive Mg^2+^ substituted P2‐Na_0.67_[Mg_0.22_Mn_0.55_ Fe_0.23_]O_2_ cathode delivering a specific capacity of ∼207 mAh g^− 1^. This robust electrochemical performance is attributed to the improved structural stability resulting from the Mg–O bonding interaction, indicating the suppressed Jahn‐Teller distortion. Upon cycling up to 150 cycles, 73% of the former capacity was maintained, pointing out stable O^2−^/O^−^ anionic redox [229].

Wang et al. constructed a pouch cell by tuning the intergrowth structure formed at desodiation of Na_0.67_Li_0.1_Fe_0.37_Mn_0.53_O_2_ to give a prominent energy density of 165 Wh kg^−1^ [227]. The peak corresponding to oxygen evolution was observed at a voltage of 4.29 V characterized by OEMS, which is in tune with the superoxo O–O stretch observed at ∼1110 cm^−1^ in in situ surface‐enhanced Raman spectroscopy (SERS). This peak is attributed to the further lattice oxidation and detrimental lattice degradation (Figure 10b,c). Li substitution into the TM site of NFMO triggers anionic oxygen redox, which is reversible at a voltage limit of 4.3 V. Highly reversible phase transformations (Figure 10d) were obtained owing to the OP4 boundary phase, which control adjacent stacking sequence that promotes structural integrity and high capacity.

Na_2∕3_Mg_1/3_Mn_2/3_O_2_ is a well‐known anionic redox material with high structural integrity during cycling. Vergnet et al. reported the impact of P2 versus O2 stacking, with P2 stacking favouring overall distortion of lattice instead of oxygen pair disproportionation, which leads to O2. Phase stability diagram for both the phases (P2 and O3) with respect to the biphasic reaction (experimental) with respect to varying Na composition is displayed in Figure 10e [228].

For x>0.66, the P2 form is thermodynamically favorable over the O2 phase, which is in accordance with the experiments. Both the phases display equal stability in the compositional range of 0.33<x<0.66. At deeper desodiation, the O2 structure is anticipated to undergo partial phase transformations into the prismatic arrangement, suggesting the existence of O–P phase that is consistent with the experimental findings [167].

### 4.4 Vacancy Based layered oxides

Introducing vacancies in the TM layers of SIB cathodes can evoke oxygen redox reactions, bolstering energy density. These defects adjust the bandgap, improve charge transfer, disrupt nearby atoms, and form more storage sites, thus promoting ion diffusion. Studies have linked vacancies in layered materials to improved electrochemical performance. Anionic redox can be initiated by introducing vacancies through the manipulation of synthesis process, such as the cooling rate and annealing duration. However, comprehending this defect chemistry alone is insufficient to create the perfect high‐capacity, reversible cathode materials [230, 231, 232, 233]. Zang et al. addressed the combined impact of strategic substitution elements and TM vacancies in the TMO_2_ layers for enhancing and stabilizing the oxygen redox chemistry in P2‐type Na_0.67_MnO_2_. Xiao et al. reported that strong Al–O bonds stabilize Mn–O bonds via Mn–O–Al sequences, preventing TM migration and limiting oxygen release. Conversely, weak Zn–O bonds cause significant O_2_ release and irreversible migration of TM layer. To evaluate how vacancy concentration in Mn affects cationic and anionic redox processes, the normalized initial charge profiles of NAMO‐NC were measured at 1 C (170 mA g^−1^) and examined across several voltage ranges, capacity retention were also studied (2.0–4.5V: 92% V and 2.0–4.0V: 98%) [234].

Na_x_[Li_y_Mn_1–y_]O_2_ cathodes having (honeycomb‐like) superstructures were studied to understand the relationships between ARR activity, cationic ordering, stability, and non‐bonding O2*p* states. Two superstructures were identified: broken (bHS‐NLMO) and intact (iHS‐NLMO) honeycomb‐like structures.

The iHS‐NLMO have de‐clustered oxygen 2*p* state, achieving an ARR‐active capacity (231 mAh g^−1^), reduced voltage drops and good cycle stability. In situ Raman spectroscopy demonstrated the stability of iHS‐NLMO is due to inhibited oxygen dimerization, which increases structural stability and prevents detrimental phase transitions (P2‐P2'), as confirmed by in situ XRD. DFT calculations indicated iHS‐NLMO to have a robust ARR structure with lower formation energy [235]. Chen et al. introduced Mg^2+^ and vacancies incorporated in the TM site of P2‐Na_2/3_[Mn_7/9_Mg_1/9_▭_1/9_]O_2_. DFT calculations and Soft X‐ray absorption spectroscopy revealed that vacancies were superior to Mg^2+^ in promoting oxygen redox at low voltages. The vacancy‐containing TMO_6_ octahedra showed asymmetry and flexibility, contributing to high structural stability. The material delivered a high capacity of 212 mAh g^−1^ with good capacity retention over 50 cycles [236]. Chen et al. designed P2‐Na_2/3_[Zn_1/9_Mn_7/9_▭_1/9_]O_2_‐P2‐NZMO‐vacancy‐containing system to investigate the vacancies not only affecting the Mg^2^^+^ spin state, but also influencing structural integrity and kinetic performance. P2‐NZMO‐Vac demonstrates exceptional capacity (>200 mAh g^−^^1^), improved rate performance, and reduced voltage hysteresis. Comprehensive analysis using different characterization techniques (temperature‐dependent magnetic susceptibility measurements, soft and hard X‐ray absorption spectroscopy (XAS), electrochemical evaluations, and DFT calculations) on NZMO‐Vac revealed that TM vacancies modify Mn^3+^ spin states, suppress Jahn‐Teller distortion, enhance structural stability, and facilitate anionic redox activity [237].

Lu et al. demonstrated P2‐Na_0.7_Mg_0.2_[Fe_0.2_Mn_0.6_▭_0.2_]O_2_ involving a reversible oxygen redox with discharge capacity of 154 mAh g–^1^. Intrinsic TM vacancies and Mg^2+^‐ions were occupied at Na sites during cycling that stabilizes structure mainly at high voltage and oxygen redox facilitated by configurations confirmed by time‐resolved techniques. Besides, initial hysteresis was due to CEI formation and structural adjustments. Mn and O ions provided charge compensation across the voltage range, while Fe redox occurred from 3.0–4.5 V [238]. Fang et al. designed P2‐Na_0.8_Mg_0.13_[Mn_0.6_Co_0.2_Mg_0.07_▭_0.13_]O_2_ with a discharge capacity of ∼176 mAh g–^1^. It featured vacancies at TM sites and presence of Mg ions in both TM and Na sites boosting flexibility and asymmetry of the TMO_6_ octahedra. The electrostatic repulsion between O–O was mitigated by adjusting the interlayer spacing with Mg^2+^ at the Na site, while the electronic structure was stabilized during charging due to the Co^3+^/Co^2+^ redox couple. The configurations ▭‐O‐▭, Na‐O‐▭, and Mg‐O‐▭ enhanced oxygen redox for charge compensation more effectively than Na‐O‐Mg, as confirmed via XAS and DFT. This material exhibited a stable voltage plateau ∼4.2 V involving oxygen redox even at high rates [239].

The Mn‐rich Na_1‐x_Li_x_ [(Mn_0.66_Co_0.17_Ni_0.17_)_0.8_▭_0.2_]O_2_ maintained a pure O3 phase during cycling, with no trace of O3‐P3 binary phase. It offered a capacity of 220 mAh g–^1^ with stable cycling. During cycling, kinetic barriers increased due to the migration of TM, creating a metastable state [240]. The P3‐Na_0.67_Co_0.2_Mn_0.8_O_2_ synthesized with varying transition metal vacancies showed promising cyclability as a positive electrode material. Synthesis using slow cooling and oxygen flow creates in‐plane vacancies, enabling reversible oxygen redox above 3.8 V without significant oxygen layer gliding. Air‐Na_0.65_Co_0.202_Mn_0.808_O_2_ exhibited an irreversible oxidation peak over 3.8 V that offers more capacity for charging. For example, Oxy‐Na_0.64_Co_0.189_Mn_0.756_O_2_ has vacancies that stabilize NaO_6_ octahedra through ▭‐Na^+^‐▭ Coulombic attraction [241].

P2‐Na_0.76_Ca_0.05_[Ni_0.23_▭_0.08_Mn_0.69_]O_2_ has been developed, comprising both cationic and anionic redox activities. The sodium layers have Ca‐ions, and TM layers has intrinsic vacancies, enhancing sodium ion mobility by disrupting Na^+^/vacancy ordering. Ca ions also act as pillars, preventing the TM layer from gliding and suppressing the P2–O2 phase transition. Reinforced Ca–O bonds prevented the lattice oxygen loss, improving anionic redox reversibility. This robust structure delivered reversible capacity of ∼153.9 mAh g^−1^. Random vacancies in the TM sites were confirmed by electrochemical measurements and physical characterizations [242].

**FIGURE 11:**
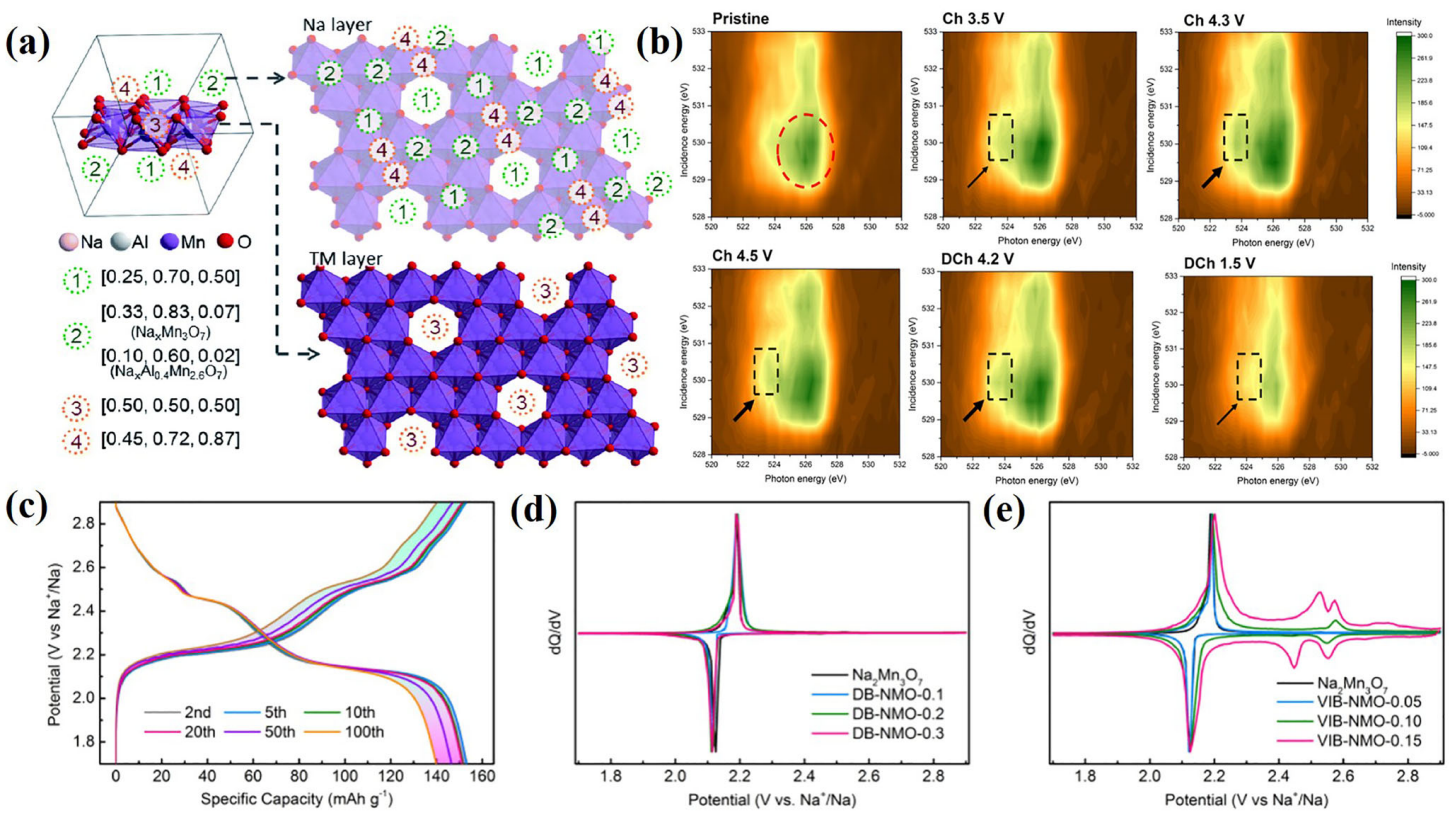
(a) The schematics illustrate the predicted sodium (Na) sites in (Na_x_Mn_3_O_7_) and Na*_x_* Al_0.4_Mn_2.6_O_7_. These diagrams provide a top view of the Na and transition metal (TM) layers within the supercells used in this study. The detailed configurations highlight the spatial distribution of Na ions within the lattice and the arrangement of transition metals. The number of occupied Na sites in the optimized structures is indicated, shedding light on the ionic occupancy and structural stability of these compounds. This visualization is critical for understanding the material's electrochemical properties and potential performance in battery applications [243]. Reprinted with permission [243] CC‐BY‐4.0 (b) O K‐edge RIXS maps at different voltages [244]. Reprinted with permission [244] CC‐BY‐4.0 (c) The charging and discharging curves of VIB‐NMO‐0.15 over 100 cycles at a rate of 0.1 C are presented. (d) For the DB‐NMO‐x samples, the differential capacity curves reveal the distinct voltage plateaus and peaks corresponding to the oxidation and reduction reactions of the active materials. (e)The rate capability of the optimal sample was thoroughly evaluated to determine its performance under varying charge and discharge rates [245]. Reprinted with permission [245] CC‐BY‐4.0, American Chemical Society.

Na_2_Mn_3_O_7_ (*P*1 space group), a layered oxide, has been studied for its high theoretical capacity of 155 mAh g^−^^1^ and its significant anionic redox contribution of 75 mAh g^−^^1^ (4.0 V vs. Na^+^/Na), resulting in high energy densities. The extra capacity arises from the unoccupied Mn site in the TM layers adjacent to the oxygen atoms of the non‐bonding O 2*p* orbitals. Based on the bonding oxygen atoms per formula unit, three oxygen are redox active during cycling. This indicates potential to increase anionic redox capacity by ∼50% if all redox‐active oxygen atoms are utilized [156, 246, 247]. By employing the modified compound Na_2.4_Al_0.4_Mn_2.6_O_7_, the effect of Al^3+^ substitution on the charge storage mechanism was studied with particular attention to structural alterations, interlayer spacing, and Na^+^ ion insertion sequences in the Na_2_Mn_3_O_7_ cathode. Oxygen release and the associated redox reactions of Mn/oxygen during cycling was examined through electrochemical analysis, structural characterization, and DFT calculations. Figure 11a shows the symmetrically inequivalent Na sites in the unit cell of Na_2.4_Al_0.4_Mn_2.6_O_7_, due to the surplus Na in Na_3_ and Na_4_, results the repulsion of Na‐ions from the Na_2_ site.[243]Hakim et al. investigated the electronic structure of Na_2_Mn_3_O_7_. XAS results (Mn LII, III‐edge) revealed that Mn remains in the Mn^4+^ state during the initial charge. Oxygen undergoes a redox reaction at high voltages as a part of charge compensation as evidenced by O K‐edge XAS and O K‐edge RIXS (Figure 11b). This oxygen redox reaction causes high polarization and sluggish sodium diffusion, as indicated by GITT tests [244]. Mg doping in Na_2_Mn_3_O_7_was examined, where 2 mol.% of Mg doping led to capacity of ∼143 mAh g^−1^. Full cells using 0.5 mol.% Mg‐doped electrode led toa promising capacity of 80 mAh g^−1^. Thus, cation doping can facilitate O^2‐/n−^ redox to reach high capacities [248]. Boron incorporation in the TM layer made the low valent Mn^3+^ electrochemically active. It led to a higher average voltage involving two redox peaks at 2.45 V and 2.55 V. This modification also weakens the O 2*p* chemical state adjacent to the Fermi level. At a high charge state such modification suppresses the overoxidation of oxygen and reinforces the layered structure during oxidation/reduction. By examining the voltage profiles and capacity retention over extended cycling, one can evaluate the efficiency, durability, and potential degradation mechanisms of VIB‐NMO‐0.15 under prolonged operational conditions (Figure 11c). No new peaks appeared in the DB‐NMO‐x (boron doped without vacancy) samples (Figure 11d). Higher boron concentration led to improved performance and rate kinetics in VIB‐NMO‐x (Na_2−3x_Mn_3_B_x_O_7_) (Figure 11e) [245].

### 4.5 Reductive Coupling for Anionic Redox

The Reductive Coupling Mechanism (RCM) refers to the interplay between cationic and anionic redox processes in electrode materials, where both TM cations and lattice oxygen anions contribute to charge compensation during battery operation. This concept has attracted significant attention in sodium‐ion batteries (SIBs) as a strategy to enhance energy density by enabling simultaneous contributions from cationic and anionic species. Traditionally, cathode capacity is limited by the number of electrons exchanged by TM cations (e.g., Mn^2+^/ Mn^3+^/ Mn^4+^); however, redox coupling allows additional charge compensation through the participation of oxygen anions, thereby increasing the overall reversible capacity [69, 249].

In practice, the interaction between cationic and anionic redox occurs dynamically during cycling. At high voltages, anionic redox processes are predominant, whereas cationic redox is more active at lower voltages [250]. With repeated cycling, lattice oxygen loss can trigger an increased contribution from TM cations to maintain charge neutrality. This shift reduces the overall anionic capacity and can lead to voltage fade, highlighting that uncontrolled interactions between oxygen and TM redox may adversely affect battery performance. By understanding and controlling this coupling, it is possible to design cathodes that maintain both high capacity and structural stability over extended cycles [251].

**FIGURE 12:**
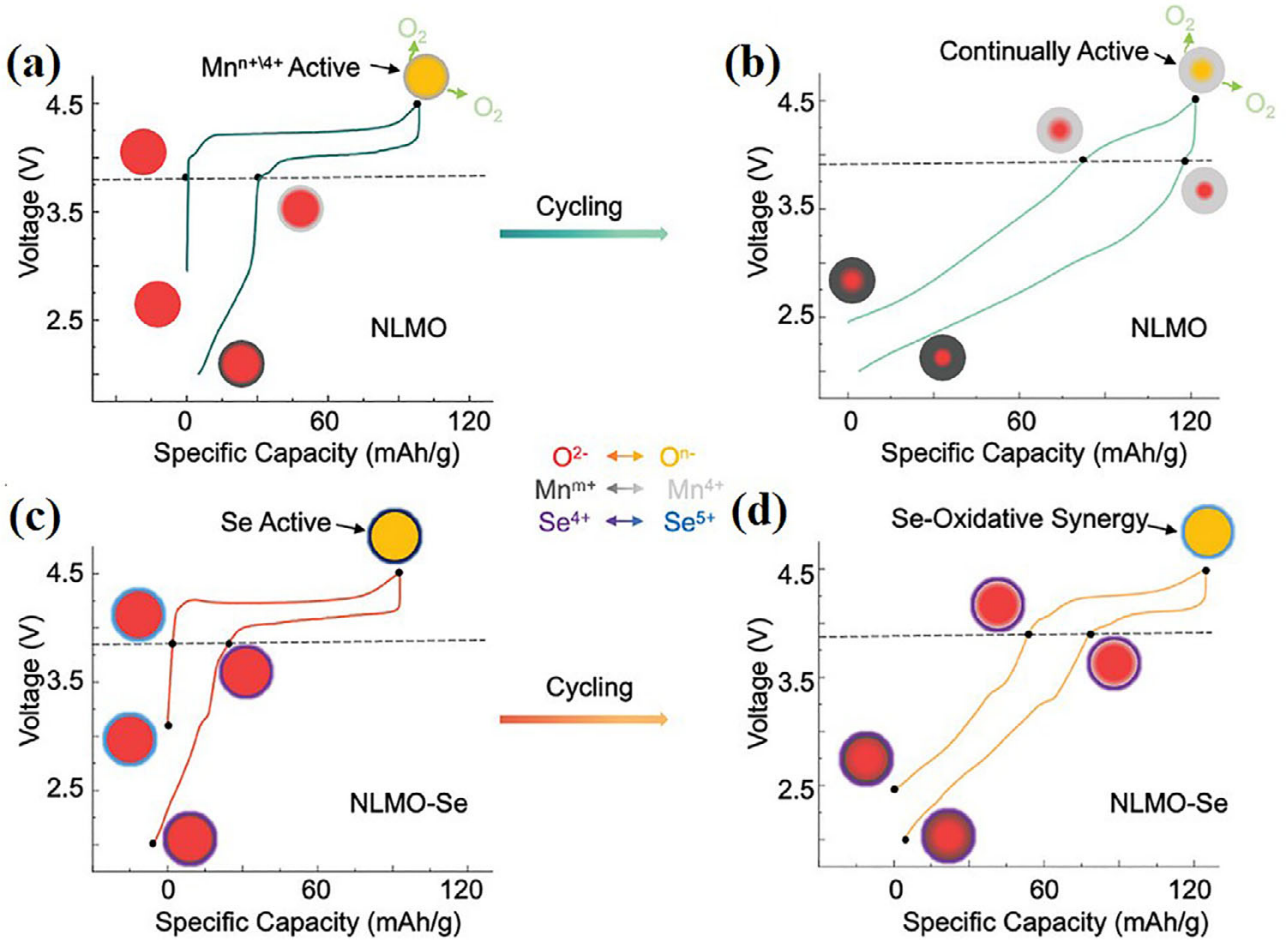
Schematic of redox couple evolution in NLMO and NLMO‐Se. (A, B) In NLMO, the Mn redox contribution increases with cycling, while the O redox contribution decreases. (C, D) In NLMO‐Se, Se dopants participate in redox reactions during charge and discharge, reducing lattice oxygen loss and Mn activation, thereby preserving oxygen anion redox activity [252]. Reprinted with permission [252] 2024 WILEY‐VCH Verlag GmbH & Co. KGaA, Weinheim.

Xue et al. showed that introducing electrochemically active selenium (Se) into Na_0.6_Li_0.2_Mn_0.8_O_2_ (NLMO) improves the reversibility of anionic redox, reduces Mn redox heterogeneity, and enhances structural stability. In NLMO, anionic redox dominates above 3.8 V, while cationic redox is active below 3.8 V (Figure 12a). During cycling, oxygen loss increases Mn participation, reducing anionic capacity and causing voltage decay (Figure 12b). Selenium doping stabilizes this coupling: the Se^4+^/Se^5+^ redox couple participates in redox, raises the energy barrier for O–O dimerization, and suppresses lattice oxygen release (Figure 12c, d). This preserves anionic redox, moderates Mn activity, balances cationic and anionic contributions, and stabilizes the voltage profile. Computational and experimental studies confirm that Se stabilizes oxygen ligands during cycling, demonstrating controlled cation–anion redox coupling. After 50 cycles, NLMO‐Se retains 84% of its anionic redox capacity, compared to 39% in pristine NLMO [252].

Studies on Co‐doped Na_2_RuO_3_ layered cathodes have shown that cobalt substitution significantly improves electrochemical reversibility compared to the pristine system. This improvement is not solely associated with enhanced cationic capacity, but rather with the role of Co as a redox mediator that facilitates the coupling between cationic and anionic redox processes. By providing an intermediate redox pathway, Co accelerates the kinetics of oxygen redox and suppresses the sluggish and irreversible behavior typically observed in Ru‐based Na‐rich oxides. As a result, the participation of lattice oxygen is strengthened while maintaining structural stability, underscoring the importance of mediator‐assisted cation–anion redox coupling in the design of high‐capacity sodium‐ion cathodes. As reported, Co‐doped Na_2_RuO_3_ delivers a high reversible capacity of 177 mAh g^−1^ (0.2 C within 1.5–4.3 V) [180]. Cu^2+^/Fe^3+^‐modified Na*_x_* Li*_y_* Mn*_1‐y_* O_2_ (NLMOs), with the composition Na_0.72_Li_0.16_Cu_0.08_Fe_0.08_Mn_0.68_O_2_ (NLCFMO), and Zn^2+^/Fe^3+^‐modified NLMO (Na_0.72_Li_0.16_Zn_0.08_Fe_0.08_Mn_0.68_O_2_, NLZFMO) illustrate the impact of cationic mediators on anionic redox processes. While both Cu and Zn can activate anionic redox, Zn‐based systems show higher oxidation peaks but suffer from poor reversibility, larger voltage hysteresis, and increased lattice oxygen loss. In contrast, Cu facilitates an RCM, where strong Cu 3*d–*O 2*p* orbital overlap enables electron transfer from oxidized oxygen to Cu ions. This mediator‐assisted interaction accelerates oxygen redox kinetics, suppresses irreversible oxygen release, and stabilizes the lattice during cycling. As a result, NLCFMO demonstrates enhanced reversible capacity (∼174 mAh g^−1^ at 0.2 C) and improved cation–anion redox coupling [253].

O3‐type layered cathodes often suffer from irreversible oxygen loss and an O3–P3 phase transition, which limits high‐voltage operation. Na_0.8_Li_0.2_Fe_0.2_Ru_0.6_O_2_ (NLFR) demonstrates a dual‐reductive coupling mechanism, where Fe and Ru ions form strong Fe/Ru–(O–O) covalent bonding at high voltages, redistributing electrons from lattice oxygen to the transition metals. This process stabilizes the oxygen redox and suppresses oxygen release. DEMS measurements confirm no detectable oxygen evolution up to 4.5 V, demonstrating the effectiveness of dual‐reductive coupling in preserving lattice oxygen [199]. Overall, these studies show that in Na‐layered oxide cathodes, cationic mediators or dual‐reductive strategies can effectively control the interplay between cationic and anionic redox, enhancing reversible capacity and stabilizing the lattice during cycling.

Table 1 summarizes representative oxygen‐redox‐based sodium layered oxide cathodes discussed in Section 4. It compiles key electrochemical parameters reported for different layered oxide systems.

**TABLE 1:** Summary of oxygen redox‐based sodium layered oxide cathodes.

Sl. No.	Cathode material	Capacity (mAh g^−1^)	Electrolyte	Voltage (V)	Retention (%)	Refs.
1	Na_1.2_Mn_0.4_Ir_0.4_O_2_	179	1 M NaClO_4_:PC+ FEC (5wt%)	1.5–44V	60% after 50 cycles	[173]
2	Na_2_Ti_0.94_Cr_0.06_O_2.97_	336 (18.9 mA g−1)	1 M NaClO_4_ in EC/PC (1:1 wt)	1.5–45V	74% after 1000 cycles	[174]
3	Na_1.14_Mn_0.57_Ti_0.29_O_2_	200	1 M NaPF_6_: PC	2.2–4.0V	—	[175]
4	Na_1.2_Mn_0.8_O_1.5_F_0.5_	172	1 M NaPF_6_ diglyme	1.5–4.5V	68% after 300 cycles	[176]
5	O‐Na_2_RuO_3_	180	1 M NaPF_6_ EC/DEC (1:1 v/v)	1.5–4.0V	89% after 50 cycles	[177]
6	Y‐doped Na_2_ZrO_3_	180	1MNaClO_4_ in EC/PC (1:1)	1.5–4.5V	97% after 50 cycles	[179]
7	Na_2_RuO_3_:Co_0.1_	177 (0.2C)	1MNaClO_4_ EC:DEC 1:1, v/v)+FEC (5wt%)	1.5–4.3V	74% after 300 cycles	[180]
8	Na_2_IrO_3_	130	1 M NaClO_4_ in EC/DMC (1:1) + FEC (1wt%)	1.5–4.0V	—	[182]
9	Na_1.5_Li_0.5_IrO_3_	190	1 M NaPF_6_ EC/DMC (1:1) + FEC (1wt%)	2.0–4.2V	80% after 50 cycles	[184]
10	Ball‐milled Na_2_SeO_3_	232	1 M NaPF_6_ PC + FEC (5vol%)	1.5–4.7V	—	[185]
11	O3‐NaLi_1/9_Ni_2/9_Fe_2/9_Mn_4/9_B_1/50_O_2_	160	1 M NaPF_6_: EC:DC(1:1)+ FEC (5wt%)	2–4.3V	82.8% after 200 cycles	[195]
12	O3‐Na(Fe_0.2_Co_0.15_Cu_0.05_Ni_0.2_Mn_0.2_Ti_0.2_)B_0.02_O_2_	108.5	1 M NaClO_4_:PC+ FEC (5wt%)	2–4.1V	95% after 100 cycles	[261]
13	O3‐Na_0.8_Ni_0.3_Fe_0.2_Mn_0.3_Li_0.1_Mg_0.02_Ca_0.05_Sb_0.03_O_2_	130	1 M NaClO_4_:EC:PC+FEC (5wt%)	2–4.2V	85% after 250 cycles	[262]
14	O3‐(Na_0.82_Mg_0.04_)(Ni_0.2_Fe_0.4_Mn_0.4_)B_0.02_O_2_	110	1 M NaClO_4_ in EC/PC/DMC (1:1:1)	2–4V	89.5% after 200 cycles	[263]
17	O3‐Na[Li_0.05_(Ni_0.25_Fe_0.25_Mn_0.5_)_0.95_]O_2_	180.1	1 M NaClO_4_:PC+ FEC(98:2)	1.75–4.4V	76% after 200 cycles	[264]
18	O3‐Na_0.826_Li_0.06_Ni_0.27_Mn_0.5_Fe_0.1_Ti_0.07_O_2_	119.8	1 M NaClO_4_:PC+ FEC (5wt%)	2.2–4.3V	81.6% after 500 cycles.	[265]
19	O3‐Na[Li_0.05_Mn_0.50_Ni_0.30_Cu_0.10_Mg_0.05_]O_2_	122.5	1MNaClO_4_ EC: DEC (1:1) +FEC (2wt%)	2–4V	81.6% after 400 cycles	[266]
20	P2‐Na_0.8_Li_0.1_Mn_0.6_Ni_0.2_Cu_0.1_O_2_	84.7	1 M NaClO_4_: PC	1.8–4V	81.7% after 400 cycles	[267]
21	P2‐Na_0.85_Li_0.12_Ni_0.22_Mn_0.66_O_2_	104.8	1 M NaClO_4_ EC:PC+FEC (5wt%)	2–4.3V	85% after 500 cycles	[268]
22	P2‐Na_0.67_Li_0.05_Ni_0.28_Mn_0.67_O_2_	144.6	1 M NaClO_4_PC+ FEC (5wt%)	2–4.25V	91.8% after 50 cycles	[207]
23	P2‐Na_0.72_Ni_0.28_Li_0.05_Mn_0.57_Ti_0.10_O_2_	120	1 M NaClO_4_ PC+ FEC (5wt%)	2.5–4.35V	80% after 200 cycles	[269]
24	P2‐Na_2/3_Ni_1/3_Mn_1/2_Ti_1/6_O_2_	152.8	1 M NaPF_6_ EC: PC (1:1) + FEC(5wt%)	2.5–4.3V	94.8% after 100 cycles	[270]
25	P2/O3‐Na_0.76_Ni_0.33_Mn_0.5_Fe_0.1_Ti_0.07_O_2_	105	1 M NaClO_4_ PC+ FEC (5wt%)	2.2–4.3V	75.4% after 500 cycles	[271]
26	P2/O3‐Na_0.67_Fe_0.3_Mn_0.5_Li_0.1_Mg_0.1_O_2_	187.8 (0.1C)	1 M NaClO_4_ EC/DEC (1:1)+ FEC (5vol%)	1.8–4.3V	96.6% after 100 cycles (1C)	[272]
27	Na_0.9_Li_0.1_Ni_0.3_Fe_0.2_Mn_0.4_Ti_0.04_Ru_0.04_Mg_0.02_O_1.9_F_0.1_	146 (C/15)	1 M NaPF_6_ PC+ FEC(2wt%)	2–4.3 V	75% after 200 cycles	[273]
28	Na_0.67_Al_1.5_Mn_0.85_O_2_	170	1 M NaPF_6_ PC+ FEC (5vol%)	2.0–4.0V	98% after 50 cycles	[234]
29	Na_2/3_[Mn_7/9_Mg_1/9_▭_1/9_]O_2_	212	1 M NaClO_4_ EC/PC/DMC (1:1:1) + FEC (2vol%)	1.5−4.5V	100% after 50 cycles	[236]
30	Na_2/3_[Zn_1/9_Mn_7/9_▭_1/9_]O_2_	204	1 M NaClO_4_ EC/PC/DMC (1:1:1) + FEC (2vol%)	1.5−4.4V	43.4% after 100 cycles	[237]
31	Na_0.7_Mg_0.2_[Fe_0.2_Mn_0.6_▭_0.2_]O_2_	155	1 M NaClO_4_ EC/PC (1:1) + FEC (5Vol%)	1.5–4.5V	71.1% after 100 cycles	[238]
32	Vacancy‐Induced Boron‐Na_2_Mn_3_O_7_	123.7	1 M NaClO_4_ EC/PC (1:1)	1.7–2.9C	89.4% after 100 cycles	[244]
33	Vacancy‐Induced ‐Na_2_Mn_2.94_Mg_0.06_O_7_	218	1 M NaPF_6_ EC:DEC(1:1)	1.5–4.5V	80.3% after 30 cycles	[248]
34	selenium‐doped Na_0.6_Li_0.2_Mn_0.8_O_2_	110	1 m NaClO_4_ PC/FEC (9:1, v/v)	2.0–4.5 V	84% after 50 cycles	[252]
35	Na_0.72_Li_0.16_Cu_0.08_Fe_0.08_Mn_0.68_O_2_	174 (0.2C)	1 M NaClO_4_ in EC/PC/DMC (1:1:1) + FEC (2 vol%)	2.0–4.5	85% after 100 cycles at 1C	[253]
36	O3‐Na_0.8_Li_0.2_Fe_0.2_Ru_0.6_O_2_	168 (10 mA g^−1^)	1 M NaClO_4_ EC/PC (1:1) + FEC (5 vol%)	1.5−4.5V	95.4% after 100 cycles	[199]
37	P2‐Na_0.67_Fe_0.5_Mn_0.45_Ru_0.05_O_2_	170 (0.2C)	1MNaClO_4_ EC:DEC 1:1, v/v) +FEC (5wt%)	2.0–4.0V	90% after 60 cycles	[274]

## 5 Challenges and Future Perspective

Recently, researchers have been investigating how to utilize anionic redox mechanisms rather than suppressing them. Utilizing anionic redox, which involves making it reversible, can improve capacity by widening the voltage window. In light of the established anionic redox mechanism, the ability to tune the relative positions of cationic and anionic levels is crucial for solid‐state chemists to discover new anionic‐redox materials.

Although anionic redox activity in layered cathode materials contributes significantly to high capacity and energy density, several challenges still hinder their practical application. (i) Voltage fade, sluggish kinetics, and voltage hysteresis remain persistent due to poor reversibility of the anionic redox reaction. (ii) Oxygen loss is triggered not only by insufficient coordination of oxygen atoms but also by intrinsic lattice instability, as observed in systems like Mg‐based P2‐Na_0.67_Ni_0.33_Mn_0.67_O_2_ and NaNi_0.5_Mn_0.5_O_2_. (iii) Doping with electrochemically inactive elements (e.g., Mg^2+^, Al^3+^, Ti^4+^) enhances structural stability but reduces theoretical capacity, highlighting the critical role of TM–O covalency and the specific redox mechanism [178, 197, 199, 220, 234, 254]. (iv) At high voltages, lattice oxygen oxidation produces unstable O_2_ dimers and superoxide species, triggering oxygen release, microstructural defects, electrolyte decomposition, and irreversible capacity loss [255, 256, 257]. (v) 4*d* (e.g., Ru, Pd, Mo) or 5*d* (e.g., Ir, W, Os) elements enhance TM–O covalency and improve anionic redox stability, their high cost and scarcity pose a substantial challenge for practical commercialization [10].

Building on these challenges, future perspectives suggest several promising directions. Harnessing reversible anionic redox remains crucial and can be achieved through cationic and anionic doping, redox‐coupling strategies involving chalcogen participation (e.g., selenium [252]) that interacts with oxygen redox chemistry and exploitation of the upper Hubbard band mechanism for dual‐band cationic‐anionic participation [50, 161, 258]. Structural design strategies such as high‐entropy cathodes, single‐crystal architectures, and rivet‐effect lattices can improve local structural diversity and suppress phase evolution from layered to spinel structures. Fluorine and boron doping are increasingly studied because their strong M–F and B–O covalent bonds suppress oxygen over‐oxidation and gas release, directly improving the reversibility of anionic redox in high‐voltage. In comparison, chalcogenides offer high reversible capacity and low voltage hysteresis, highlighting complementary routes for designing high‐performance sodium layered cathodes [161]. Interface and electrolyte engineering, including the use of high‐voltage stable electrolytes and optimized surface coatings (e.g. Al_2_O_3_, TiO_2_, phosphates, polymers), can mitigate side reactions and further enhance reversibility [259, 260]. Finally, advanced characterization techniques, particularly operando Raman and multi‐modal approaches, are essential to monitor both bulk and surface anionic redox processes in real time, guiding rational design of next‐generation high‐energy, stable, and reversible Na‐ion cathodes. Collectively, these strategies highlight a multi‐pronged future direction, integrating materials design, electronic tuning, and interface control, which will accelerate the development of advanced anionic‐redox‐based energy storage systems.

**FIGURE 13:**
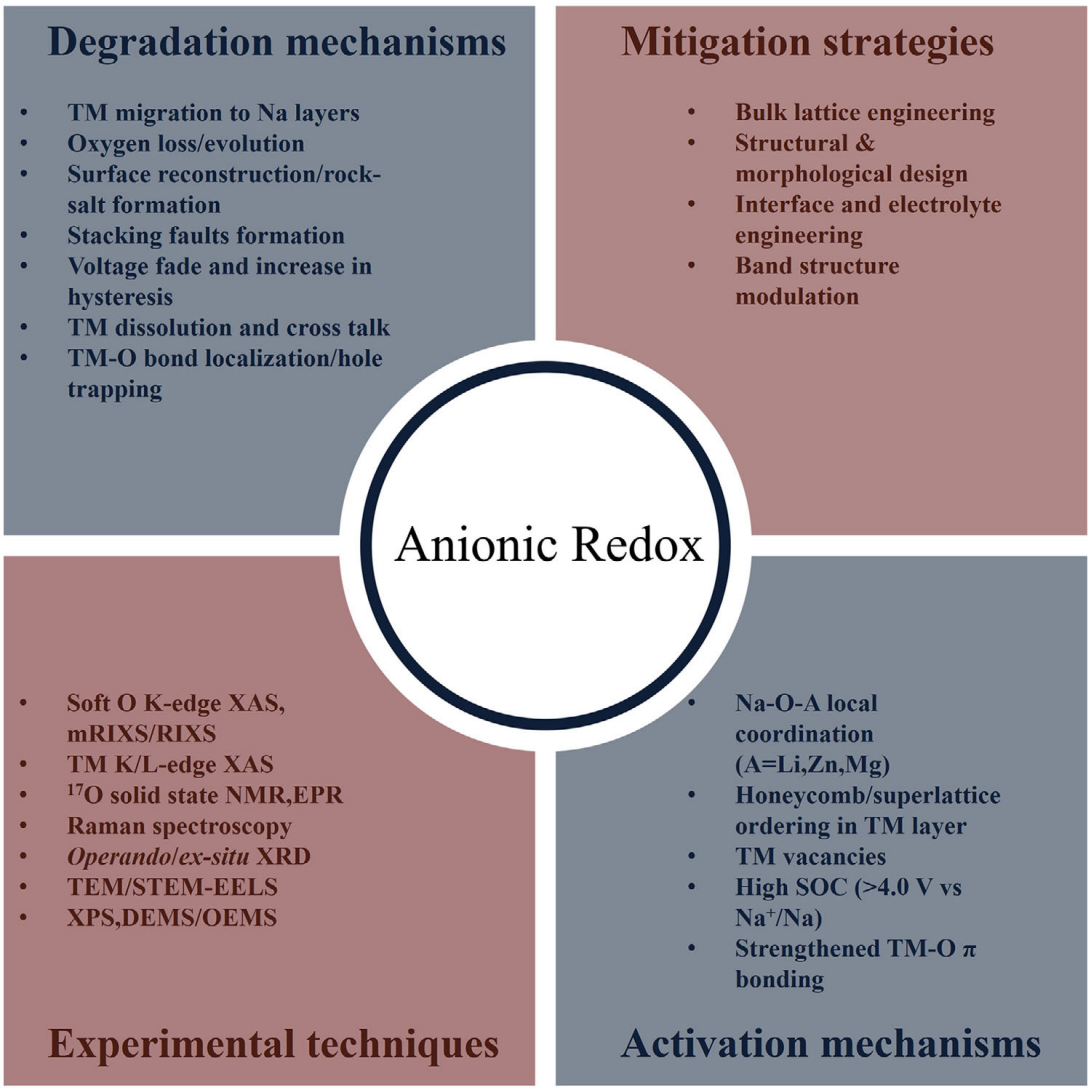
Overview of anionic redox chemistry in layered oxide cathodes, summarizing the central features discussed in this review.

Figure 13 presents a concise schematic of anionic redox in layered oxide cathodes. The figure brings together activation mechanisms, commonly used characterization techniques, major degradation pathways, and representative mitigation strategies, providing a unified view of how anionic redox influences cathode stability and performance.

## 6 Conclusions

The future of Na‐ion batteries (SIBs) lies in the optimization and full exploitation of redox coupling between cationic and anionic processes. The incorporation of anionic redox couples provides a pathway to significantly enhance energy density by enabling multi‐electron transfer mechanisms. These developments hold immense potential to revolutionize energy storage technologies, delivering high‐capacity, economic, and environmentally friendly solutions. However, a major drawback of these cathode materials is their inherent structural instability, which hinders their practical application in real‐world technologies. To mitigate or eliminate these structural instabilities, several strategies have been proposed. One promising approach involves enhancing the covalency of the metal‐oxygen (M–O) bonds. This method aims to stabilize the structure by strengthening the interactions between metal and oxygen atoms, thereby reducing the propensity for the structural degradation that typically accompanies high energy densities in these materials. To develop further understanding, various characterization methods, including XAS, mRIXS, ND, and EELS, are used to study anionic oxygen redox reactions. Notably, mRIXS is particularly effective due to its reduced sensitivity to TM 3*d* states. Consequently, advanced and innovative characterization techniques should be adopted alongside traditional electrochemical methods for a comprehensive analysis of the anionic redox mechanism. These studies aim to elucidate how the oxidation and reduction of anions, in conjunction with cationic redox reactions, could enhance the performance and capacity of next‐generation Na‐ion batteries. Various studies have made significant contributions to the understanding of anionic redox chemistry in both Na‐ion and Li‐ion battery materials. This interdisciplinary approach, bridging knowledge and materials between different battery systems, has the potential to accelerate the development of advanced energy storage solutions with improved performance and sustainability.

## Funding

DST SERB Start‐up Research Grant (SRG/2022/001301), Humboldt Fellowship, Deutsche Forschungsgemeinschaft (DFG, German Research Foundation) under Germany´s Excellence Strategy‐EXC 2154‐Project number 390874152.

## Conflicts of Interest

The authors declare no conflicts of interest.
